# Novel method of transpulmonary pressure measurement with an air-filled esophageal catheter

**DOI:** 10.1186/s40635-021-00411-w

**Published:** 2021-09-17

**Authors:** Paul Bernard Massion, Julien Berg, Nicolas Samalea Suarez, Gilles Parzibut, Bernard Lambermont, Didier Ledoux, Pierre Pascal Massion

**Affiliations:** 1 Department of Intensive Care, University Hospital of Liege, Sart-Tilman B35, 4000 Liege, Belgium; 2 Department of Anesthesiology, University Hospital of Liege, Sart-Tilman B35, 4000 Liege, Belgium; 3 Division of Allergy, Pulmonary and Critical Care Medicine, Vanderbilt University Medical Center, Nashville, TN 37232 USA

**Keywords:** Esophageal pressure, Esophageal balloon catheter, Pleural pressure, Transpulmonary pressure, Acute respiratory distress syndrome, Lung-protective ventilation, Ventilator-induced lung injury, Patient self-inflicted lung injury, Respiratory mechanics

## Abstract

**Background:**

There is a strong rationale for proposing transpulmonary pressure-guided protective ventilation in acute respiratory distress syndrome. The reference esophageal balloon catheter method requires complex in vivo calibration, expertise and specific material order. A simple, inexpensive, accurate and reproducible method of measuring esophageal pressure would greatly facilitate the measure of transpulmonary pressure to individualize protective ventilation in the intensive care unit.

**Results:**

We propose an air-filled esophageal catheter method without balloon, using a disposable catheter that allows reproducible esophageal pressure measurements. We use a 49-cm-long 10 Fr thin suction catheter, positioned in the lower-third of the esophagus and connected to an air-filled disposable blood pressure transducer bound to the monitor and pressurized by an air-filled infusion bag. Only simple calibration by zeroing the transducer to atmospheric pressure and unit conversion from mmHg to cmH_2_O are required. We compared our method with the reference balloon catheter both ex vivo, using pressure chambers, and in vivo, in 15 consecutive mechanically ventilated patients. Esophageal-to-airway pressure change ratios during the dynamic occlusion test were close to one (1.03 ± 0.19 and 1.00 ± 0.16 in the controlled and assisted modes, respectively), validating the proper esophageal positioning. The Bland–Altman analysis revealed no bias of our method compared with the reference and good precision for inspiratory, expiratory and delta esophageal pressure measurements in both the controlled (largest bias −0.5 cmH_2_O [95% confidence interval: −0.9; −0.1] cmH_2_O; largest limits of agreement −3.5 to 2.5 cmH_2_O) and assisted modes (largest bias −0.3 [−2.6; 2.0] cmH_2_O). We observed a good repeatability (intra-observer, intraclass correlation coefficient, ICC: 0.89 [0.79; 0.96]) and reproducibility (inter-observer ICC: 0.89 [0.76; 0.96]) of esophageal measurements. The direct comparison with pleural pressure in two patients and spectral analysis by Fourier transform confirmed the reliability of the air-filled catheter-derived esophageal pressure as an accurate surrogate of pleural pressure. A calculator for transpulmonary pressures is available online.

**Conclusions:**

We propose a simple, minimally invasive, inexpensive and reproducible method for esophageal pressure monitoring with an air-filled esophageal catheter without balloon. It holds the promise of widespread bedside use of transpulmonary pressure-guided protective ventilation in ICU patients.

**Supplementary Information:**

The online version contains supplementary material available at 10.1186/s40635-021-00411-w.

## Background

There is a strong rationale for proposing transpulmonary pressure (P_L_)-guided protective ventilation in acute respiratory distress syndrome (ARDS) [1–4]. The ARDS lung is modeled in two regions, one consolidated and collapsed, responsible for the impairment of oxygenation, and one functional region called “baby lung”. This latter region is also inflamed, responsible for the ARDS mechanical characteristics, and at risk for evolving ventilator-induced lung injury (VILI) [5, 6] and patient self-inflicted lung injury [7]. To limit global lung stress, lung-protective ventilation starts by limiting tidal volume (Vt) and thereby airway driving pressure (ΔP) [8], with greater mortality benefit recently demonstrated in ARDS patients with higher driving pressures and respiratory system elastance values [9]. However, this airway pressure (Paw) approach does not take into account altered chest wall elastance (Ecw) [10], often increased in critically ill patients with extrapulmonary ARDS [11]. It also neglects various transpulmonary pressures (P_L_) known to trigger the VILI during both inspiration and expiration. During passive condition, static driving P_L_ (ΔP_L_) is the real global distending force stressing the lung [12]. End-expiratory P_L_ (P_L_ee) and elastance-derived end-inspiratory P_L_ (P_L_ei,_ER_) are key determinants of atelectrauma in mid-to-dependent zones and of barotrauma/volutrauma in non-dependent zones, respectively. During assisted ventilation, i.e., in active condition, pleural pressure swing and transpulmonary pressure swing (ΔP_L_dyn) are also indexes of inspiratory effort and dynamic lung stress, respectively [13]. Hence, to optimize protective ventilation and to quantitatively confirm its protective settings, taking lung stress, respiratory effort, potential dyssynchrony, Ecw and lung elastance (El) into account [1, 14], we need to monitor both airway and pleural pressures.

Esophageal pressure (Pes) is a well-known surrogate of the pleural pressure [3]. The difference between airway and Pes indicates P_L_, while the esophageal pressure swing (ΔPes) divided by Vt and the ΔP_L_ divided by Vt are valid estimates of Ecw and El, respectively [3]. Therefore, a simple, inexpensive, accurate, and reproducible method of measuring esophageal pressure reflecting pleural pressure would greatly facilitate protective ventilation in the intensive care unit. While the esophageal balloon catheter method requires a semi-invasive 10-cm-long balloon catheter, complex in vivo calibration [15], clinical expertise and expensive specific equipment, our new methodology greatly simplifies the approach.

## Methods

An adapted air-filled esophageal open catheter method without balloon, using a disposable catheter and transducer allows for reproducible esophageal pressure measurements, without any specific material requirements.

### Description of the air-filled esophageal catheter method

**Fig. 1:**
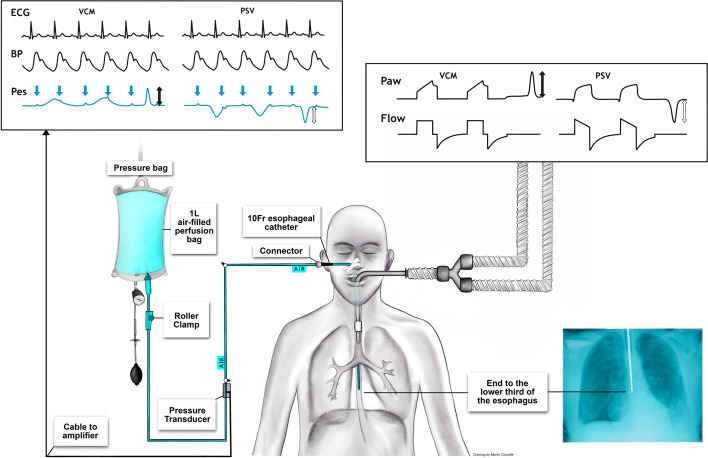
Air-filled catheter without balloon method. The esophageal catheter is placed at the lower-third of the esophagus, connected to an air-filled transducer bound to the monitor and pressurized by an air-filled perfusion bag. The three ways for controlling positioning are cardiac arteficts (blue arrows on Pes trace), esophageal-to-airway pressure change ratio (under passive—black arrows and active condition—white arrows) and X-rays projection of guidewire (extremity at the lower-third of the esophagus)

Here, we use a disposable low compliance polyvinyl esophageal suction catheter, originally intended for oral, nasopharyngeal or tracheobronchial suctioning, 49 cm long, 10 Fr and 3.3–2.0 mm outer–inner diameters. As shown in Fig. 1, the catheter is connected to an air-filled disposable blood pressure transducer bound to the monitor. A 1-l saline infusion bag is emptied, backfilled with air and pressurized at 100 mmHg by a pressure infusion bag with manometer (Additional file 1: Figure S1) and connected to the air-filled intravenous set, so that the transducer delivers a continuous air flow rate of ~ 2.5 ml/min. The pressurized system guarantees open-ended catheter patency, undamped values and signal stability. Air-labeled flags are disposed along the air-filled pressure line for safety. To facilitate the nasal or oral placement of the esophageal catheter and its visualization on chest X-rays, a siliconized guide wire of nasogastric enteral feeding tube is temporarily inserted in the catheter and bended to match the desired length. The catheter is positioned first in the stomach. Proper gastric position is assessed by auscultation of a 10-mL air flush and, after connection to the transducer by observation of positive deflections on waveform during inspiration or when gentle stomach compressions are imposed. The catheter is withdrawn to the lower-third of the esophagus until esophageal waveform is confirmed by small cardiac artifacts and spontaneous inspiratory negative deflections. Appropriate position of the catheter is confirmed in three ways: (i) by chest X-rays with the guide wire (Additional file 2: Figure S2); (ii) by visualization of cardiac artifacts on the esophageal waveform, and (iii) by equivalent changes in esophageal and airway pressures during the dynamic end-expiratory occlusion test maneuver. In passive breathing condition, gentle external chest compressions are performed during expiratory occlusion. In active breathing condition, spontaneous efforts occur against occlusion (Baydur’s maneuver) (Additional file 3: Figure S3). During the occlusion test, an esophageal-to-airway pressure change ratio (ΔPes/ΔPaw) close to unity (± 10–20%) validates the technique as an adequate estimate of pleural surface pressure and thereby the proper position of the catheter [3, 16].

Before any esophageal measurement, the open-ended catheter is flushed with 3 ml of air using a syringe in order to remove any distal secretion. The transducer is zeroed at atmospheric pressure. Values are recorded in mmHg and converted in cmH_2_O.

Esophageal catheter subocclusion by secretions is suspected when abrupt, vertical falls, staircase steps or increasing slopes disrupt the esophageal pressure wave (Additional file 4: Figure S4). Deobstruction of the catheter requires a flushing procedure to restore a proper signal.

For clinical purpose, we measure end-inspiratory and end-expiratory pressures in both airway and esophagus, during controlled and assisted modes. This allows computation of transpulmonary pressures in order to apply an optimal transpulmonary-guided lung-protective ventilation (Additional file 3: Figure S3). To facilitate bedside calculations we have designed an online transpulmonary pressures calculator [17]. For more details on the method, see the complete standard operating procedure in the Additional file 9.

### Comparison with the reference balloon catheter

The esophageal balloon catheter used in our study was the 14 Fr multifunction nasogastric feeding catheter NutriVent™ (Sidam, Italy). We connected the balloon catheter via a rigid air-filled line to a disposable and locked pressure transducer bound with the monitor. In vivo calibration, including end-expiratory and end-inspiratory pressure–volume curves for different balloon volumes following Mojoli et al. [15] was performed and allowed the determination of the optimal filling volume of the esophageal balloon (Vbest), the esophageal wall elastance (Eew) and the pressure generated by the esophageal wall (Pew). Calibrated values of Pes (Pes minus Pew corresponding to Vbest) were not computed here. Additional ex vivo comparison between air-filled catheter and balloon catheter is presented in the online supplement (Additional file 5: Figure S5; Additional file 9).

### Population

We studied 15 consecutive critically ill patients requiring prolonged mechanical ventilation (> 48 h), without contraindications such as coagulation, hemodynamic, esophageal or gastric disorder or recent cardiothoracic surgery considering the need for external chest compression during the occlusion test. Our methodological monocentric study was conducted in a tertiary 50-bed ICU university hospital in Liege, Belgium. All patients in passive breathing condition were sedated, paralyzed and ventilated in volume-controlled mode in semi-recumbent position. In active condition, all patients were ventilated in pressure support mode by a Servo-i Maquet respirator (Getinge, Sweden) that allows end-expiratory occlusion in this mode.

### In vivo comparison of both esophageal pressure methods

All esophageal pressure measurements via the air-filled catheter and the balloon catheter were performed sequentially rather than simultaneously, to avoid catheter interference on the esophageal wall and potential bias. Air-filled catheter was withdrawn in the upper-third of esophagus during balloon catheter measurements. Inversely, balloon was deflated during air-filled catheter measurements, but without removal of the nasogastric feeding balloon catheter since the air-filled catheter is supposed to be used in ventilated patients usually requiring feeding catheter. Random order of reading was applied. Twelve measurements were performed repeatedly by three different observers, namely sequentially 3 series for each of them plus 3 supplemental series for the first observer. Each series of esophageal measurements consisted in six specific timepoints, i.e., during dynamic external chest compression, inspiratory occlusion and expiratory occlusion in the controlled mode (Additional file 3: Figure S3A), and during Baydur’s maneuver, inspiratory deflection and end-expiratory measure in the assisted mode (Additional file 3: Figure S3B).

### Direct comparison of esophageal pressure with pleural pressure

Two patients (#7 and #8) presented right pleural drain that allowed pleural pressure measurements via fluid-filled regular pressure transducer. Recordings of simultaneous esophageal and pleural pressures traces together with ventilatory parameters could be performed using Philips IntelliVue MP70 with spirometry module and flow sensor. High-definition 500-Hz recordings required to analyze signal stability and frequency components of the pressure signals were performed using I-Care Pro Software (PLHealthcare & eSense, Belgium) connected to an MP70 interface. The frequency spectra were obtained by applying fast Fourier transforms (Python with NumPy package, Python Software Foundation) to each esophageal and pleural pressure signals, extracted from a 80-s multiparameter period recording, according to the Welch’s method [18].

### Statistical analysis

Continuous variables are presented as mean ± standard deviation (range minimum–maximum) and qualitative variables as count (%). The agreement between the reference balloon catheter method (Balloon) and the air-filled catheter method (Catheter) was assessed using the Bland–Altman technique adapted for repeated measurements analysis [19–21]. Repeatability and reproducibility of esophageal pressure measurements were evaluated by intra-observer and inter-observer intraclass correlation coefficients. Paired t-tests were used to compare paired measurements of continuous variables in the patients' sample. P-value < 0.05 was considered significant. Statistics were performed using Stata (StataCorp 2021, College Station, TX) and RStudio (RStudio Team 2020, Boston, MA) software.

## Results

### Clinical data

Three hundred thirty-six series of measurements were performed in the 15 ICU patients requiring prolonged mechanical ventilation included in the study. All patients were studied in volume-controlled mode (180 series), and 13 of them in pressure support mode (156 series). Table 1 reports their demographic and respiratory variables.

**Table 1:** Demographic and respiratory parameters of patients included in the study

Variables (*n* = 15)	Values
Age (yr)	55.8 ± 8.8 (34–66)
Male (%)	10 (66)
Body mass index (kg/m^2^)	28 ± 7 (17–40)
Obesity (%)	6 (40)
Diagnosis:	
ARDS SARS-CoV-2 (%)	6 (40)
Neurological injury (intracerebral hemorrhage, stroke, post-anoxic) (%)	6 (40)
Non-ARDS pulmonary injury (COPD, pneumonia) (%)	3 (20)
Extracorporeal membrane oxygenation (%)	2 (13)
SAPS III	60 ± 11 (45–84)
Parameters in volume-controlled mode (*n* = 15):	
Tidal volume (ml/kg predicted body weight)	6.1 ± 1.3 (2.7–7.4)
Plateau pressure (cmH_2_O)	19 ± 4 (12–25)
PEEP (cmH_2_O)	7 ± 3 (5–10)
Respiratory rate (min^−1^)	19 ± 5 (15–34)
PaO_2_/FiO_2_ (mmHg)	172 ± 88 (63–380)
PaCO_2_ (cmH_2_O)	52 ± 15 (34–92)
Respiratory system elastance (cmH_2_O/(ml/kg))	1.8 ± 1.2 (0.9–5.5)
Parameters in pressure support (*n* = 13):	
PEEP (cmH_2_O)	5 ± 1 (5–10)
Inspiratory pressure (cmH_2_O)	8 ± 3 (2–12)
Respiratory rate (min^−1^)	18 ± 4 (13–25)
PaO_2_/FiO_2_ (%)	184 ± 53 (81–267)
PaCO_2_ (mmHg)	46 ± 10 (34–67)

ARDS, adult respiratory distress syndrome; SARS-COV-2, severe acute respiratory syndrome—coronavirus-2; COPD, chronic obstructive pulmonary disease; SAPS, Simplified Acute Physiology Score; PEEP, positive end-expiratory pressure

### Methodological characteristics

While the patient’s nasal–tragus–xiphoid distance was 51 ± 3 cm (45–54), correct positioning of the esophageal catheter was obtained with its outer part (from nostril to funnel) of 10 ± 3 cm (5–15) and of the balloon catheter at 43 ± 3 cm (40–50).

In vivo calibration of the balloon catheter determined an optimal balloon volume of 2.5 ± 0.5 ml (1.5–3), esophageal wall elastance of 1.1 ± 0.4 cmH_2_O/L and pressure generated by the esophageal wall of 1.8 ± 0.9 mmHg that could theoretically be subtracted from esophageal pressures to obtain calibrated values (not done). Complete air-filled esophageal catheter implementation (including calibration) took 7.3 ± 1.9 min, versus 26 ± 7.4 min for the balloon catheter (*p* < 0.001).

Pressurization of the air-filled circuit was mandatory for accurate, undamped and stable esophageal pressure signal. While closing the system (by the roller clamp or complete disconnection) induced dampening and instability of the signal within a few minutes, inversely opening the roller of the pressurized air-filled circuit induced an increase of the values of ~ 4 (2–6) cmH_2_O and stabilization of the amplitude for more than 20 min. Subocclusion of the air-filled catheter occurred occasionally, but our flushing procedure usually succeeded in deobstruction (Additional file 3: Figure S3). Catheter replacement was only required twice and after several days in two cases of profusely secreting patients. Since any residual esophageal air was re-aspirated before measurement and pressurized air-filled circuit was removed between each measurement procedure, no patients accumulated excessive esophageal air nor presented gastric air accumulation. Spontaneous esophageal contractions, hiccup reflex, cough reflex as well as the gradual withdrawal of the esophageal catheter interfered with esophageal pressure waveforms.

Ex vivo comparison of the air-filled catheter and the balloon catheter using pressure chambers found exactly equal esophageal and inner chamber pressure changes (Additional file 5: Figure S5).

### In vivo comparison of both methods included several aspects

**Fig. 2:**
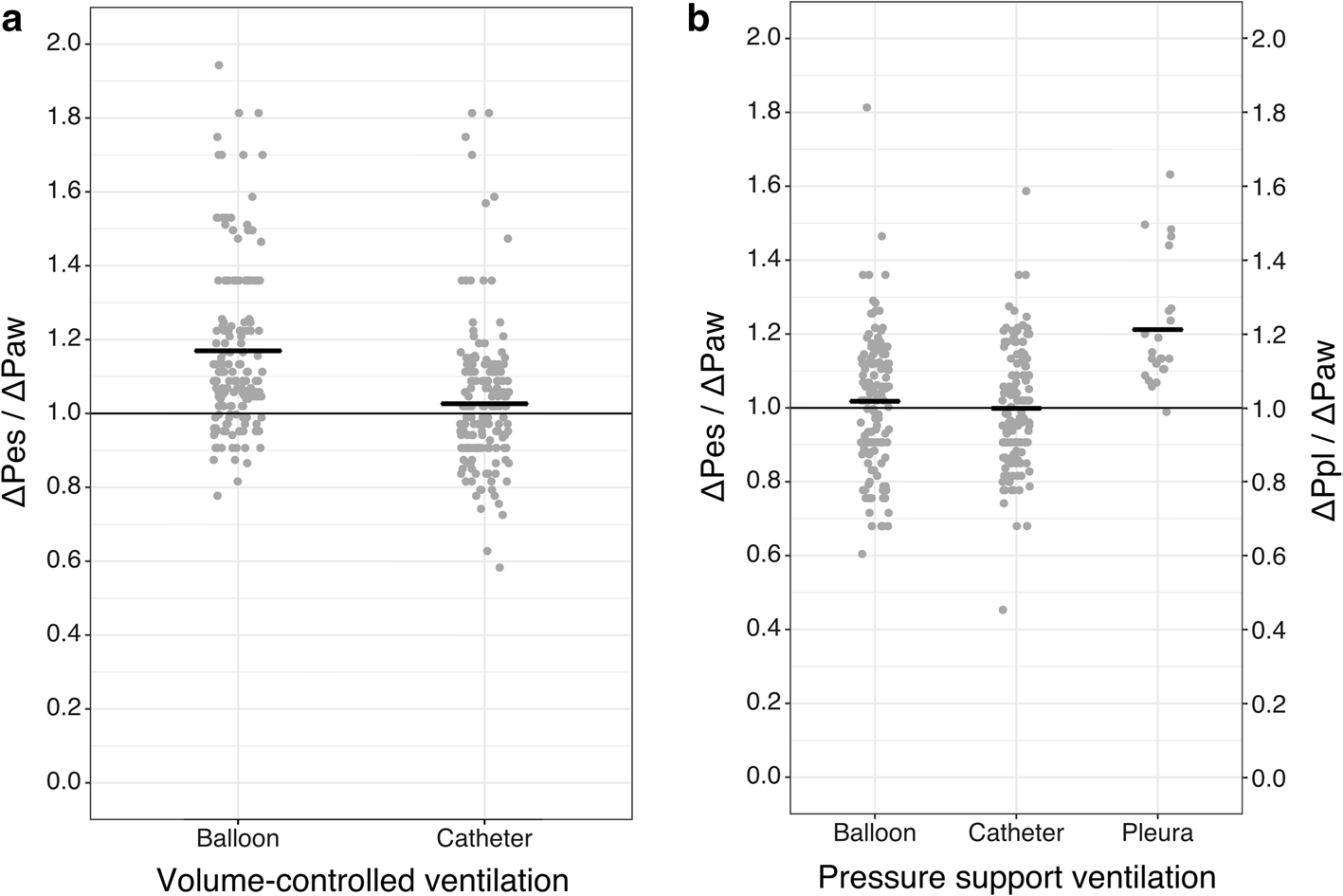
Esophageal-to-airway pressure change ratios in controlled and assisted modes. Esophageal-to-airway pressure change ratios: **a** in volume-controlled mode, induced by external chest compressions during end-expiratory occlusion (dynamic positive pressure occlusion test), using both balloon (left) and catheter (right) method; **b** in assisted mode, induced by spontaneous inspiratory efforts against airway occlusion test (Baydur’s maneuver). For comparison in **b**, pleural-to-airway pressure change ratios obtained in patients with pleural pressure measurements. Black lines indicate means of ratios

First, esophageal-to-airway pressure changes ratios after external chest compression in passive condition were close to one for the air-filled catheter method (1.03 ± 0.19, *n* = 180) and less optimal for the balloon method (1.17 ± 0.21, *n* = 180). In active condition, during Baydur’s maneuver, both methods obtained ratios close to one (1.00 ± 0.17, *n* = 141 and 1.02 ± 0.18, *n* = 144, respectively; see Fig. 2). In our two patients with pleural drainage, 6-min-long simultaneous pleural and esophageal pressure recordings showed a larger amplitude of change in pleural pressure compared to the change in esophageal pressure (balloon: ΔPpl = 14.1 ± 1.7 vs. ΔPes_B = 11.9 ± 1.0 cmH_2_O, ΔPpl/ΔPes_B ratio = 1.21 ± 0.11; catheter: ΔPpl = 14.1 ± 1.7 vs. ΔPes_C = 11.7 ± 1.4 cmH_2_O; ΔPpl /ΔPes_C ratio = 1.19 ± 0.11).

Second, in the controlled mode, we observed good repeatability (intra-observer) and reproducibility (inter-observers) for the measurements obtained with the air-filled catheter methods both for plateau pressures (repeatability 0.89 [0.76; 0.96] and reproducibility 0.89 [0.76; 0.96]) and end-expiratory pressures (0.90 [0.81; 0.96] and 0.89 [0.76; 0.96]). In the assisted mode, repeatability and reproducibility of end-expiration pressures measurement were excellent too (0.96 [0.92; 0.99] and 0.99 [0.96; 1.00]) (Additional file 6: Figure S6). We obtained similar repeatability and reproducibility for these same pressures measurements with the balloon method (all between 0.86 [0.71; 0.95] and 0.91 [0.84;0.97]).

**Fig. 3:**
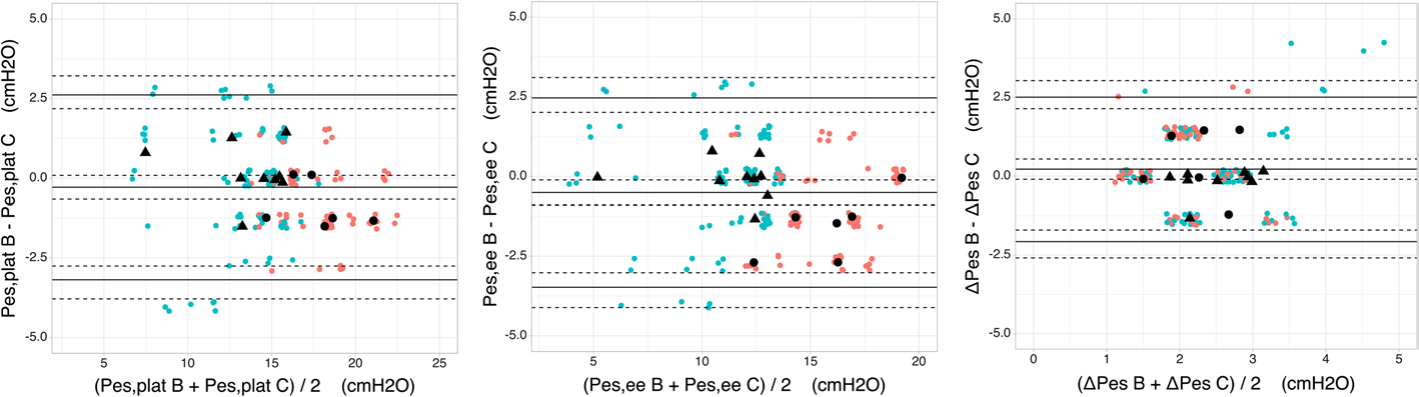
Bland–Altman analyses in obese and non-obese patients under controlled mode. The difference between the plateau (left), end-expiratory (middle) and delta (right) esophageal pressures measured by the air-filled catheter and the balloon catheter in volume-controlled mode are plotted against the mean of the two measurements. Solid lines represent the mean differences and the limits of agreement. Dashed lines represent their respective 95% confidence interval. The colored circles represent single measurements (*n* = 12 for each patient), with non-obese patients (*n* = 9) in green and obese ones (*n* = 6) in red. Black triangles and circles represent the medians of all measurements in non-obese and obese patients, respectively

Third, Bland–Altman analyses revealed that, compared to the balloon reference method, our method was accurate—with no systematic bias—and precise as shown by narrow limits of agreement, LoA). This was observed without drift over the entire range of esophageal pressure measurements (Spearman’s correlation coefficient rho not significantly different from zero). In the controlled mode (Fig. 3), methods were comparable for plateau pressure (bias −0.3 [95% confidence interval, CI −0.7; 0.1] cmH2O; LoA: −3.2 to 2.6 cmH_2_O; rho = −0.1 [95% LA: −0.4; 0.2]), end-expiratory pressure (bias −0.5 [95% CI −0.9; −0.1] cmH_2_O; LoA −3.5 to 2.5 cmH_2_O; rho = −0.1 [−0.4; 0.1]) and delta pressure (bias 0.2 [95% CI −0.1; 0.5] cmH_2_O; LoA −2.1 to 2.5 cmH_2_O; rho = 0.2 [95% CI −0.1; 0.4]). Similar results were obtained in the subset of obese patients (body mass index of 35.7 ± 2.3) with a bias of −0.7 [95% CI −2.8; 1.4], −1.1 [95% CI −1.7; −0.5] and 0.4 [95% CI −0.4; 1.1], respectively, for the same pressures. In the assisted mode (Additional file 7: Figure S7), we also observed limited bias for all three measurements, with a better precision for end-expiratory pressure (bias −0.2 [95% CI −0.8; 0.5] cmH_2_O; LoA −3.7 to 3.4 cmH_2_O; rho = 0.0 [95% CI −0.5; 0.4]) than for peak inspiratory deflection (bias 0.1 [95% CI −2.0;2.3] cmH_2_O; LoA −9.7 to 9.9 cmH_2_O; rho = 0.1 [95% CI −0.1; 0.3]) and esophageal pressure swing (bias −0.3 [95% CI −2.6; 2.0] cmH_2_O; LoA −10.3 to 9.8 cmH_2_O; rho = 0.2 [95% CI −0.1; 0.4]).

**Fig. 4:**
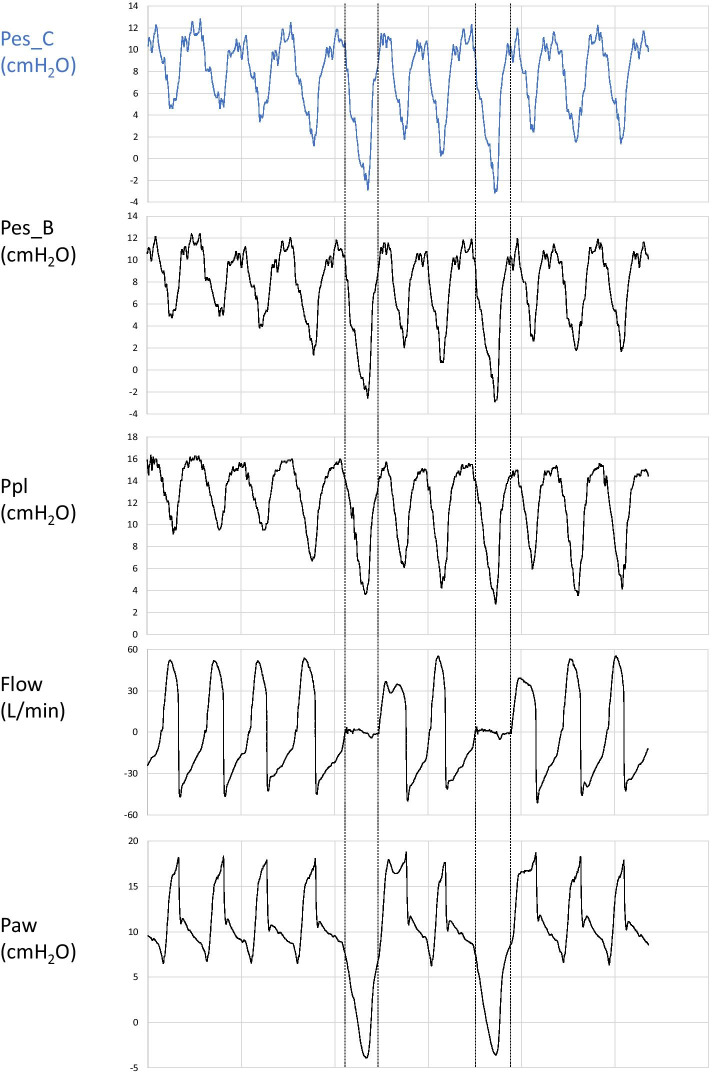
Representative simultaneous esophageal and pleural pressures waveforms. Simultaneous esophageal and pleural pressure traces are recorded from air-filled esophageal catheter (Pes_C), esophageal balloon catheter (Pes_B) and pleural catheter (Ppl), together with airway pressure (Paw) and Flow traces in a patient in assisted mode. Two successive end-expiratory occlusion tests induce increased esophageal, pleural and airway pressure deflections and are delimited by vertical lines

**Fig. 5:**
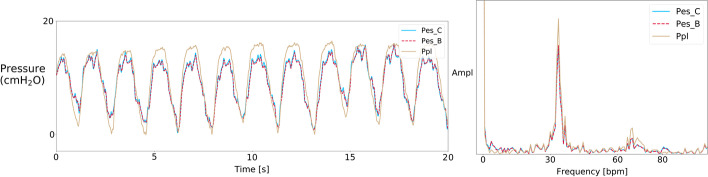
Power spectral analysis of esophageal and pleural pressure signals. The frequency spectra (right) and esophageal pressure signals (left) of both air-filled catheter and balloon catheter revealed concordant predominant respiratory component (large peak at 33 bpm) but also cardiogenic noise from heart rate component (second peak at 80 bpm). Pleural pressure curve and superposition of the three curves with their spectra are shown

Fourth, direct in vivo comparison of both esophageal pressures with pleural pressure curves confirmed the reliability of air-filled catheter esophageal pressure to estimate pleural pressure (Figs. 4, 5). As an example, Cheyne–Stokes traces illustrated the same concordance of signals (Additional file 8: Figure S8). We obtained the frequency spectra of the esophageal curves by applying fast Fourier transform (Fig. 5). Respiratory spectrum was the predominant component of both esophageal pressure curves, followed by the cardiogenic noise of heart rate spectrum. To assist the clinician in applying transpulmonary pressure-guided lung-protective ventilation, we have designed a dedicated online esophageal pressure calculator for both active and passive ventilation conditions [17].

Finally, the cost of our method was limited; close to €18 for one complete disposable circuit against €183 for the reference method.

## Discussion

The use of an air-filled esophageal catheter without balloon to measure esophageal pressure was only reported twice, to detect accidental esophageal intubation [22], and by adapting a balloon catheter to measure esophageal pressure in pigs [23]. We describe here for the first time a minimally invasive method allowing stable, repeatable, reproducible, and accurate esophageal pressure measurements, using a pressurized air-filled circuit consisting of disposable materials, and requiring only a simple calibration and flushing procedure, independently of any specific ventilator ports. We successfully validated our method through an ex vivo and an in vivo comparison with the reference esophageal balloon catheter method. We obtained good repeatability and good reproducibility of our measurements in both controlled and assisted ventilatory modes. The Bland–Altman analyses confirmed the absence of bias of our method compared to the reference balloon method. The fast Fourier transform revealed that the air-filled catheter-derived esophageal pressure and balloon esophageal pressure provided a measurement with little noise as shown by the largest amount of the power concentrated at respiratory rate frequencies, which is concomitant of pleural pressure signal.

Of note, the increased pleural-to-airway pressure change ratio around 1.2 during the Baydur occlusion test was probably related to the approximately 20% higher amplitude of pleural pressure change compared to esophageal pressure. This larger pleural swing has already been reported and may be attributed to both the juxta-diaphragmatic location of pleural catheters and the liquid method of pleural pressure measurement.

The balloon method requires thick and expensive material, time-consuming and sophisticated in vivo calibration [15] and clinical expertise. In contrast, our air-filled esophageal catheter method is minimally invasive, inexpensive, rapid, simple, immediately available, and accurate.

Furthermore, proper position of the air-filled catheter is easier to find compared to the balloon catheter. Indeed, due to its smaller pressure transmitting area, the air-filled catheter is more responsive to pressure changes and hence, more closely follows pressure changes when withdrawing the catheter from the stomach to the retrocardial esophageal position, which allows easy detection of cardiac artifacts that indicate adequate positioning. Replacement of the catheter is much easier thanks to its small size and its natural tendency to follow the nasogastric feeding tube when inserted alongside. If correctly secured to the nose, the catheter stays in proper position, and remains functional and stable for several hours (i.e., ~ 12 h) as long as the pressurized infusion bag kept inflated and at 100 mmHg of pressure. Subocclusion of the catheter by secretions is the main potential disadvantage compared with the secretion-protected balloon catheter. This occurs mainly in profusely secreting patients, i.e., 2–7 days after the catheter placement. Nevertheless, this problem is usually solved by the flushing procedure, and rarely requires catheter change. In our hands, the size of the 10 Fr catheter corresponds to the best compromise between the risk of subocclusion by secretions (increased with the 8 Fr) and the invasiveness and the air leakage rate through the pressurized circuit (increased with the 12 Fr).

Our method provides a reliable surrogate of pleural pressure in both passive and active breathing conditions. In passive breathing condition, elastance-derived end-inspiratory, end-expiratory and driving P_L_ were recently validated as key pressures responsible for barotrauma/volutrauma in non-dependent zones [13], atelectrauma in mid-to-dependent zones [13] and the global lung stress [14], respectively. In active breathing condition, peak end-inspiratory P_L_, esophageal pressure swing and transpulmonary pressure swing provide valid estimates of inspiratory stress, inspiratory effort and dynamic lung stress [1, 14], respectively. Our method, together with our online calculator, may help to adapt ventilator settings towards potential therapeutic targets from recent recommendations [2, 4, 14, 24], by modifying PEEP, Vt or inspiratory pressure accordingly.

The limitations of our methodological study are its monocentric design, the small size of our population and the absence of healthy volunteers. The next step will require multicentric validation on a larger cohort of patients. A major limitation of the study is that to avoid catheter interferences, the comparison between the air-filled catheter and the balloon catheter was performed at two different, although very closed, timepoints, which had an impact on simultaneity of measurement of transpulmonary pressure dynamic changes in assisted mode. Hence, the lower precision for peak and delta esophageal pressure measurements in assisted mode is mainly explained by the sequential rather than simultaneous design and by the variability of spontaneous inspiratory efforts. Even if esophageal pressure changes are valid estimates of pleural pressure changes, absolute esophageal pressure values should also be interpreted with caution. Using a high-resolution manometry catheter, a recent Swedish study [25] demonstrated a high variability of esophageal pressures along the esophagus, depending on complex interactions between the patient’s position, the patient’s lung and chest wall mechanics, the concerned part of the esophagus as well as mediastinal weight and cardiac compression.

## Conclusions

In summary, we propose a simple, inexpensive, and reproducible tool for esophageal pressure monitoring using an air-filled esophageal catheter without balloon. It holds the promise of widespread bedside use of transpulmonary pressure-guided protective ventilation in patients with ARDS.

## Supplementary Information

**Additional file 1: Figure S1.** Air-filled circuit assembly. The 1L saline infusion bag is emptied and backfilled with air through the pressure transducer using a 50-ml syringe (A), then pressurized with a pressure infusion bag (B) with a manometer at 100 mmHg (C).

**Additional file 2: Figure S2.** Chest X-rays of patients with the air-filled esophageal catheter. Conventional anteroposterior (left) and lateral (right) chest X-rays display the extremity (black arrow) of the air-filled esophageal catheter (containing its guide wire) at the third lower part of the esophagus in two patients without (upper panel) and with (lower panel) balloon catheter (white arrow). Intensity of X-rays dose is specified.

**Additional file 3: Figure S3.** Air-filled esophageal catheter-guided ventilation in controlled and assisted modes. A. In passive condition, illustrative waveforms of blood pressure (BP), esophageal pressure (Pes, in mmHg), air flow, airway pressure (Paw, in cmH_2_O) and volume (Vol). After zeroing and subsequent end-inspiratory and end-expiratory occlusions, three sternal compressions (white arrows) induce equivalent increases in esophageal and airway pressures. B. Same waveforms in active condition. In spontaneous breathing, dynamic end-expiratory occlusion test induces two equivalent esophageal and airway depressions (white arrows). *ΔP* driving pressure, *ΔPdyn* dynamic driving pressure, *ΔPes* esophageal pressure swing, *ΔP*_*L*_ driving transpulmonary pressure*, ΔP*_*L*_*dyn* dynamic transpulmonary pressure swing, *ECG* electrocardiogram*, PEEP* positive end-expiratory pressure*, PEEPtot* total PEEP, *Pes,ee* end-expiratory Pes, *Pes,i* inspiratory Pes, *Pes,plat* plateau Pes, *P*_*L*_*ee* end-expiratory transpulmonary pressure, *P*_*L*_*ei,*_*ER*_ elastance-derived end-inspiratory transpulmonary pressure, *P*_*L*_*peak* peak transpulmonary pressure, *Ppeak* peak airway pressure, *Pplat* plateau airway pressure.

**Additional file 4: Figure S4.** Subocclusion of the air-filled esophageal catheter and flushing procedure. Abrupt vertical falls, staircase steps or increasing slopes in the esophageal pressure wave (A to D) indicate subocclusion by secretions. Flushing 3 ml of air (E, F) or 10 ml of air (G) or pulling out for 2 cm (H) enables deobstruction in most cases.

**Additional file 5: Figure S5.** Ex vivo comparison of air-filled catheter and balloon catheter pressure transductions. A. Ex vivo comparison of air-filled catheter pressure (Pes_C), balloon catheter pressure using a 4 ml balloon volume (Pes_B) and inner chamber pressure (Pch) during 5 ml of air increments from 0 to 50 ml, injected and then removed by the chamber port (right) of the small pressure chamber (left). B. Ex vivo comparison of the same pressures (right), in a larger pressure chamber containing a test lung (left) allowing for 100 ml to 200 ml tidal volume inflations. Two successive (end-inspiratory and end-expiratory) pauses are performed. Note that volume curves underestimate true volume inflation due to tubing distension.

**Additional file 6: Figure S6.** Repeatability and reproducibility of air-filled esophageal catheter measurements. Variability of plateau (*upper panel*) and end-expiratory (*lower panel*) esophageal pressures measured using the air-filled catheter in 15 patients under volume-controlled ventilation. *Left*, intra-observer variability (repeatability) showing six repeated measurements (colored) by the same observer plotted against their medians (black). *Right*, inter-observer variability (reproducibility) showing medians from three different observers A, B and C (colored) plotted against the medians from all observers (in black: triangles for non-obese and circles for obese). Lines of equality are shown.

**Additional file 7: Figure S7.** Bland–Altman analysis in patients under assisted mode. The difference of the peak (left), end-expiratory (middle) and delta (right) esophageal pressure measurements by the air-filled catheter and the balloon methods in assisted mode are plotted against the mean of the measurements. Solid lines represent the mean differences and the limits of agreement. Dashed lines represent their respective 95% confidence interval. The colored circles represent single measurements (*n* = 12 for each patient), with non-obese patients (*n* = 8) in green and obese ones (*n* = 5) in red. Black triangles and circles represent the medians of all measurements in non-obese and obese patients, respectively.

**Additional file 8: Figure S8.** Esophageal pressures curves during Cheyne–Stokes respiration. Simultaneous esophageal pressures are recorded with the air-filled esophageal catheter and the balloon catheter in one patient with spontaneous Cheyne–Stokes respiration. Note the increase in expired CO_2_ before starting polypnea in the lower panel due to active expiration at the end of the respiratory pause (arrow).

**Additional file 9. ** The complete Standard Operating Procedure of the air-filled esophageal catheter method and the ex vivo comparison of both esophageal pressure methods are presented.

## Data Availability

The datasets used and analyzed during the current study are available from the corresponding author on reasonable request.

## Abbreviations

ARDS: Acute respiratory distress syndrome
ΔP: Airway driving pressure
ΔPaw: Change in airway pressure
ΔPdyn: Dynamic airway driving pressure
ΔPes: Change in esophageal pressure, esophageal pressure swing
ΔPes/ΔPaw: Esophageal-to-airway pressure change ratio
ΔP_L_: Driving transpulmonary pressure, change in transpulmonary pressure
ΔP_L_dyn: Dynamic change in transpulmonary pressure, transpulmonary pressure swing
ΔPpl: Change in pleural pressure, pleural pressure swing
ΔPpl/ΔPaw: Pleural-to-airway pressure change ratio
Ecw: Chest wall elastance
El: Lung elastance
ER: Elastance ratio of lung to respiratory system
Ers: Respiratory system elastance
Paw: Airway pressure
PEEP: Positive end-expiratory pressure
PEEPtot: Total PEEP
Pes: Esophageal pressure
Pes,ee: End-expiratory esophageal pressure
Pes,i: Inspiratory esophageal pressure
Pes,plat: Plateau esophageal pressure
P_L_: Transpulmonary pressure
P_L_ee: End-expiratory transpulmonary pressure
P_L_ei,_ER_: Elastance-derived end-inspiratory transpulmonary pressure
P_L_peak: Peak end-inspiratory transpulmonary pressure
Ppeak: Peak airway pressure
Pplat: Plateau airway pressure
SARS-CoV-2: Severe acute respiratory syndrome corona virus 2
