# Supplementary figures and images for: Novel method of transpulmonary pressure measurement with an air-filled esophageal catheter
Source: Intensive Care Med Exp. 2021 Sep 17;9:47. doi: 10.1186/s40635-021-00411-w (PMC8445653; doi:10.1186/s40635-021-00411-w)

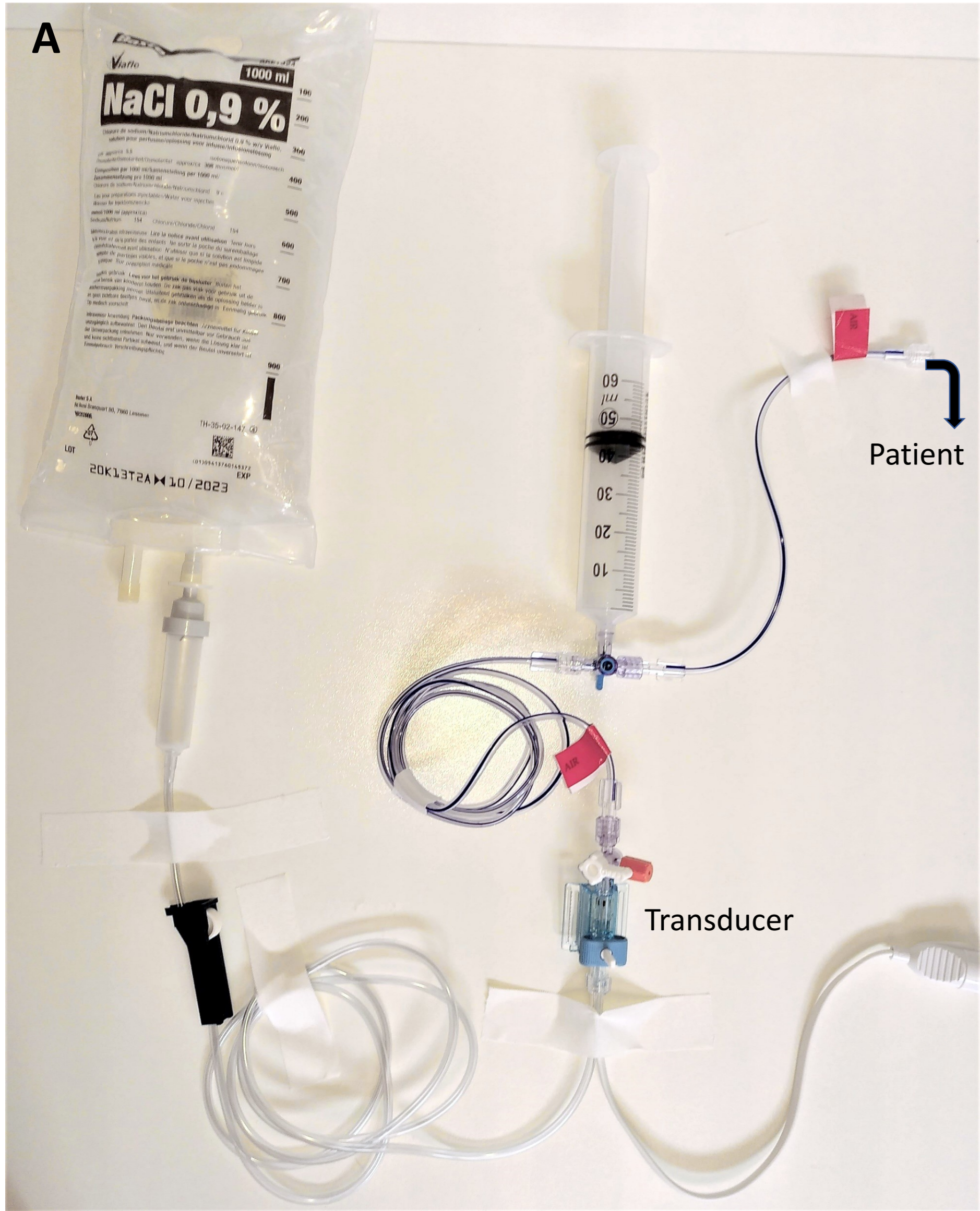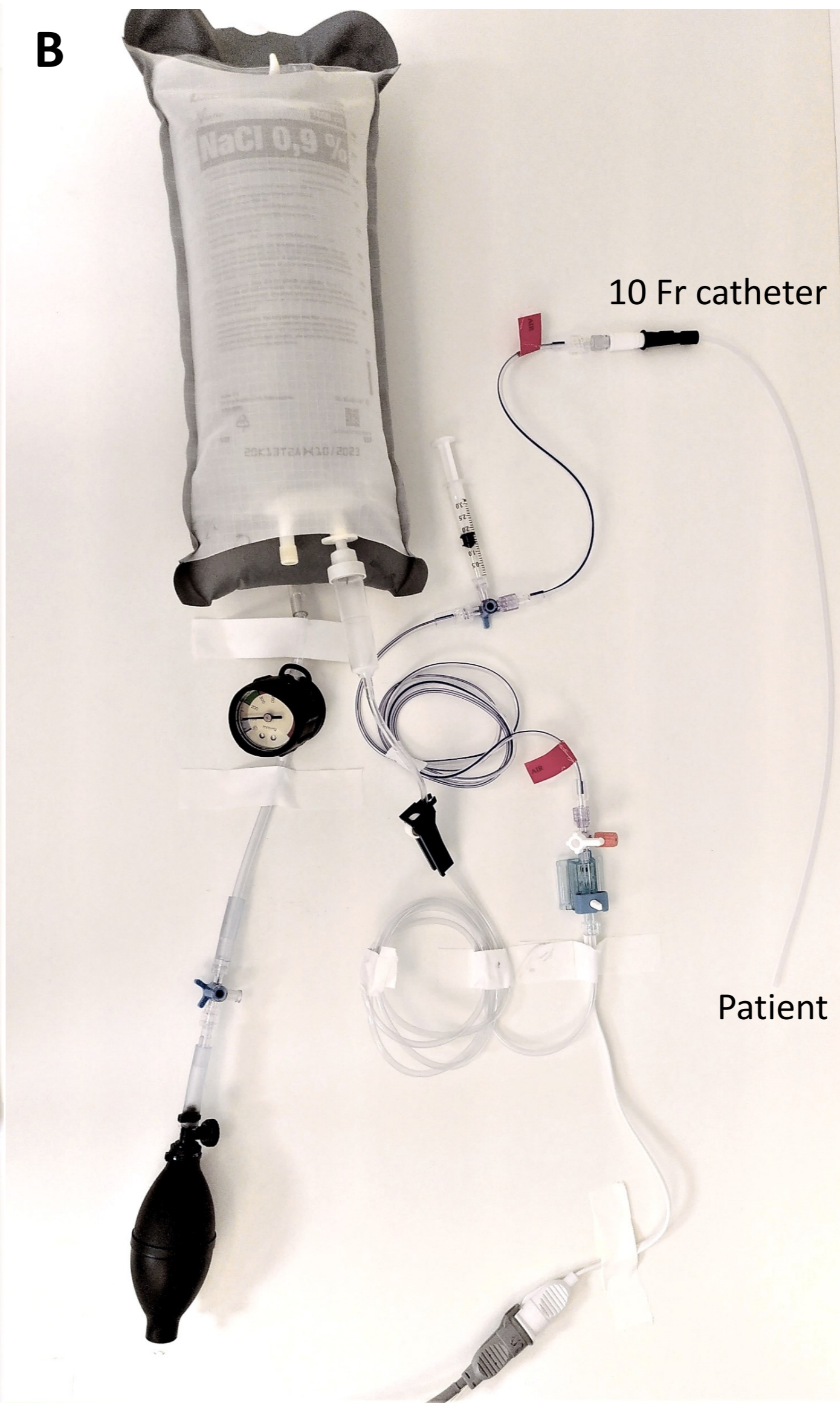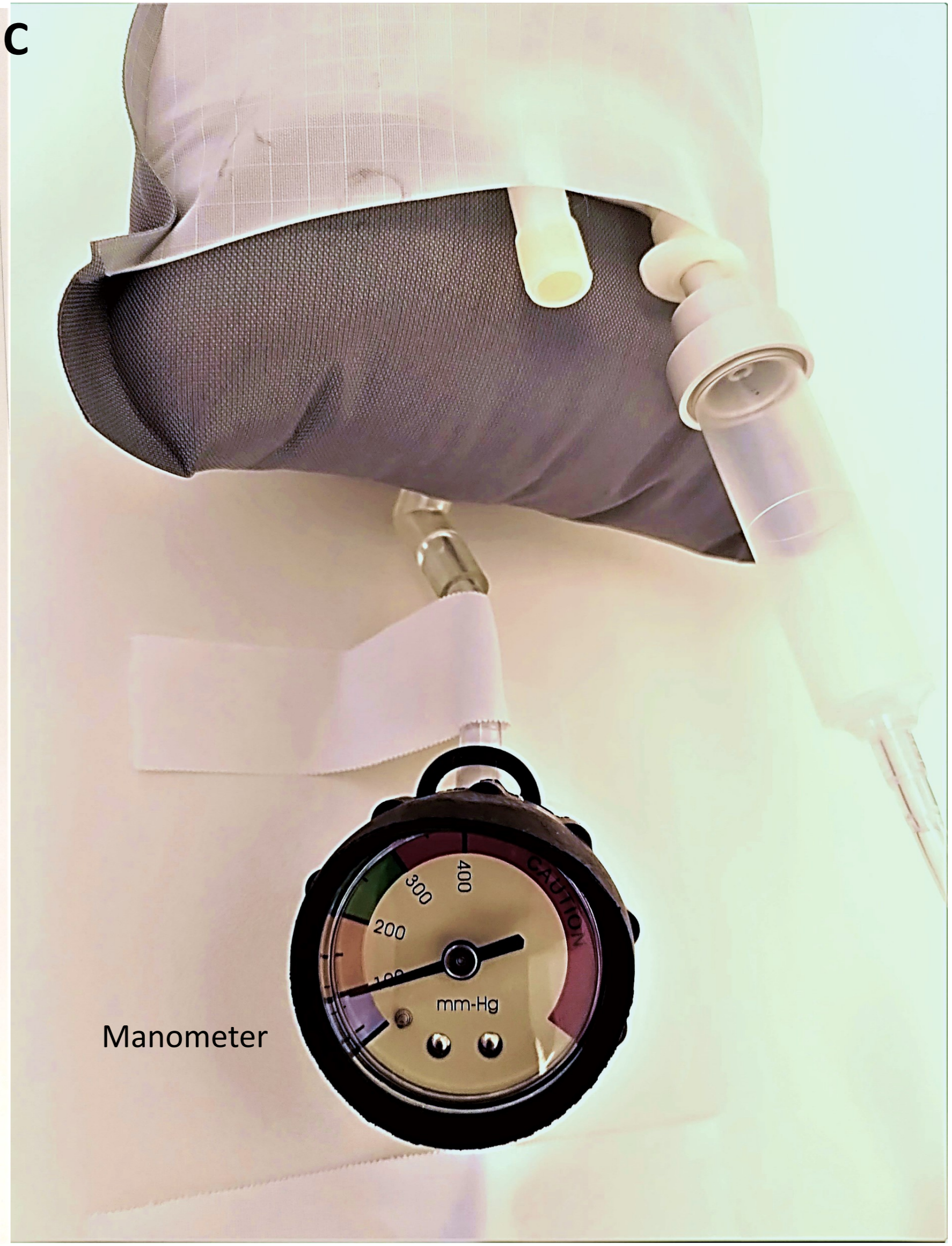

Supplement: Supplementary file 1 — Additional file 1: Figure S1. Air-filled circuit assembly. The 1L saline infusion bag is emptied and backfilled with air through the pressure transducer using a 50-ml syringe (A), then pressurized with a pressure infusion bag (B) with a manometer at 100 mmHg (C). [file 40635_2021_411_MOESM1_ESM.pdf]

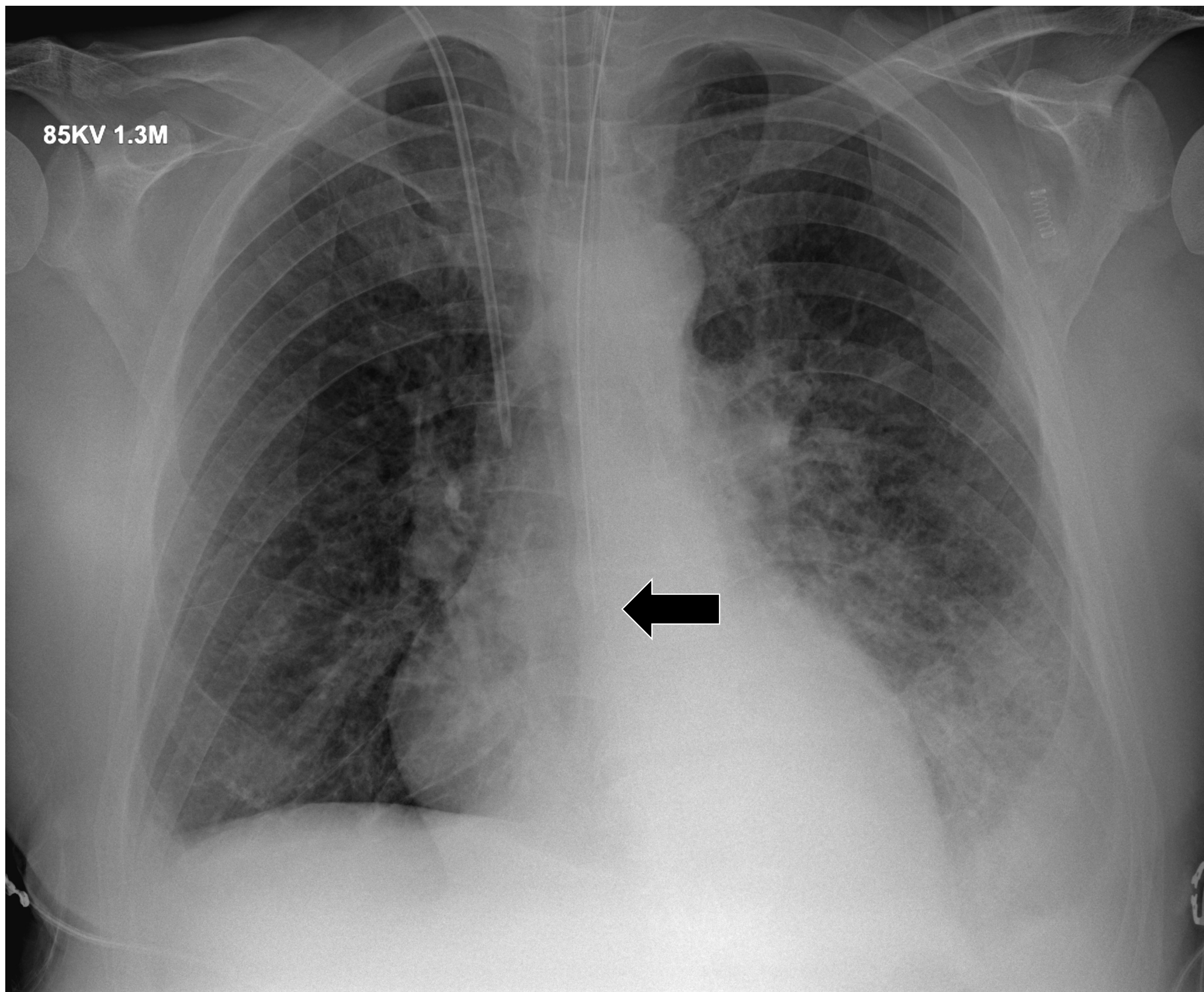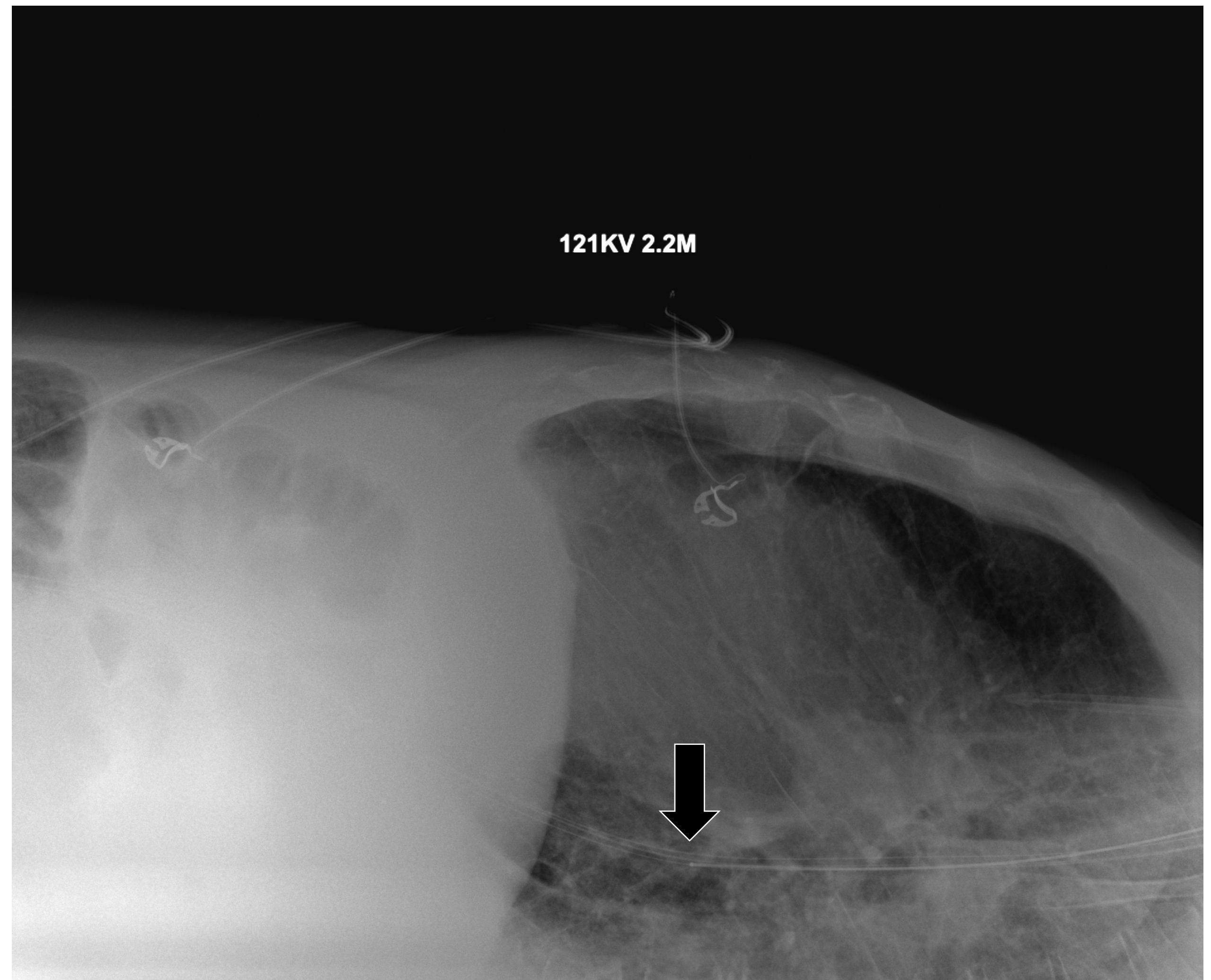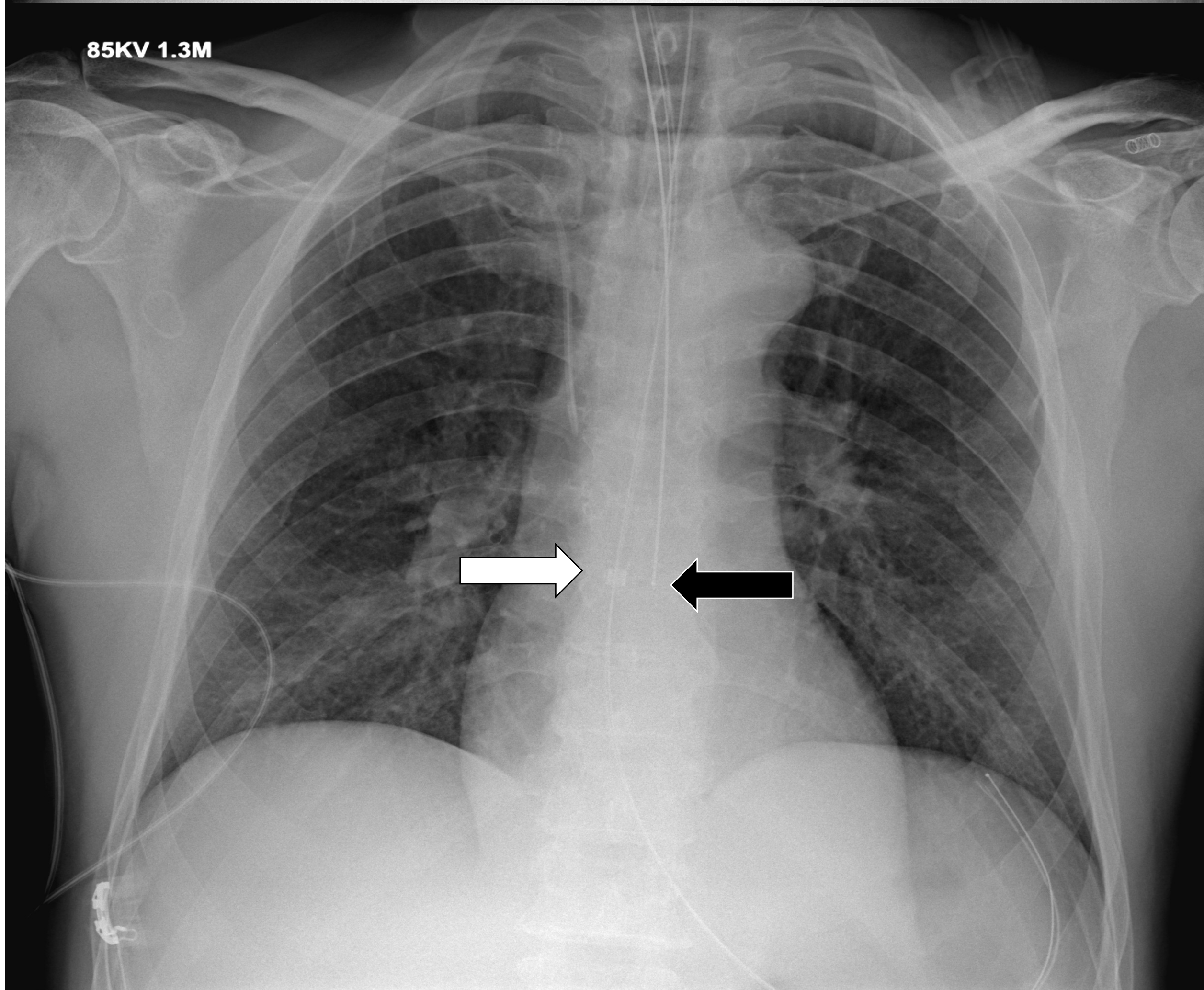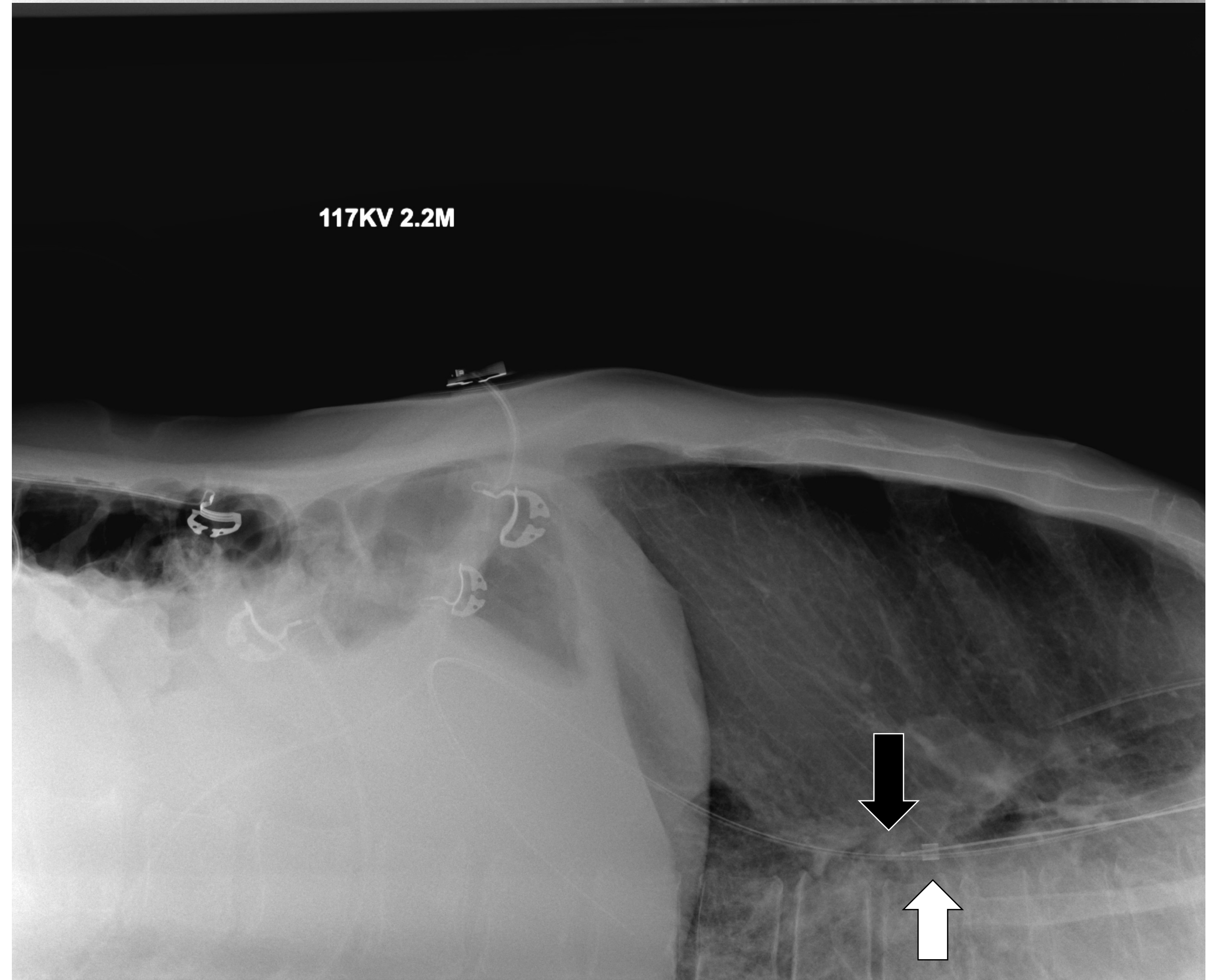

Supplement: Supplementary file 2 — Additional file 2: Figure S2. Chest X-rays of patients with the air-filled esophageal catheter. Conventional anteroposterior (left) and lateral (right) chest X-rays display the extremity (black arrow) of the air-filled esophageal catheter (containing its guide wire) at the third lower part of the esophagus in two patients without (upper panel) and with (lower panel) balloon catheter (white arrow). Intensity of X-rays dose is specified. [file 40635_2021_411_MOESM2_ESM.pdf]

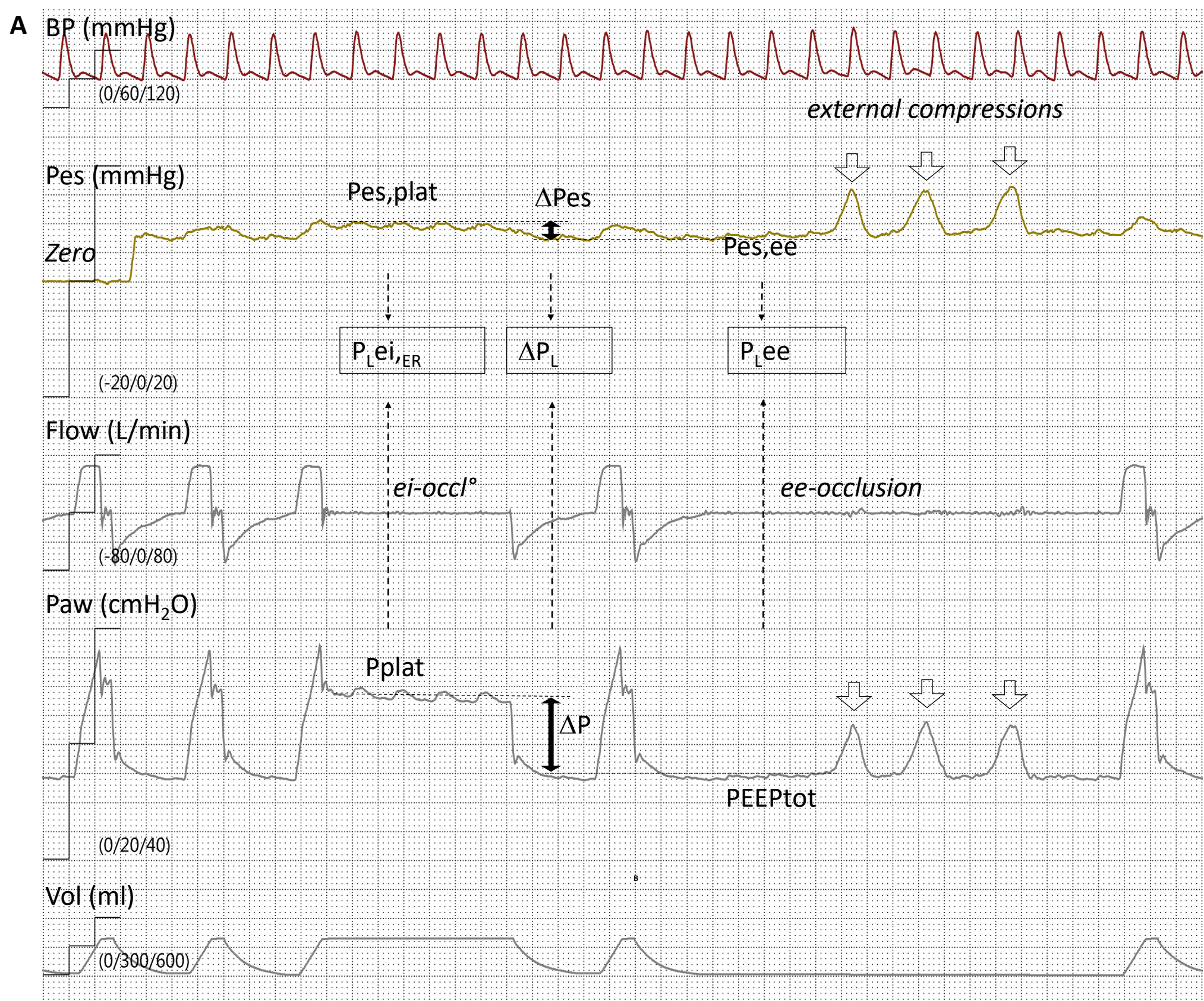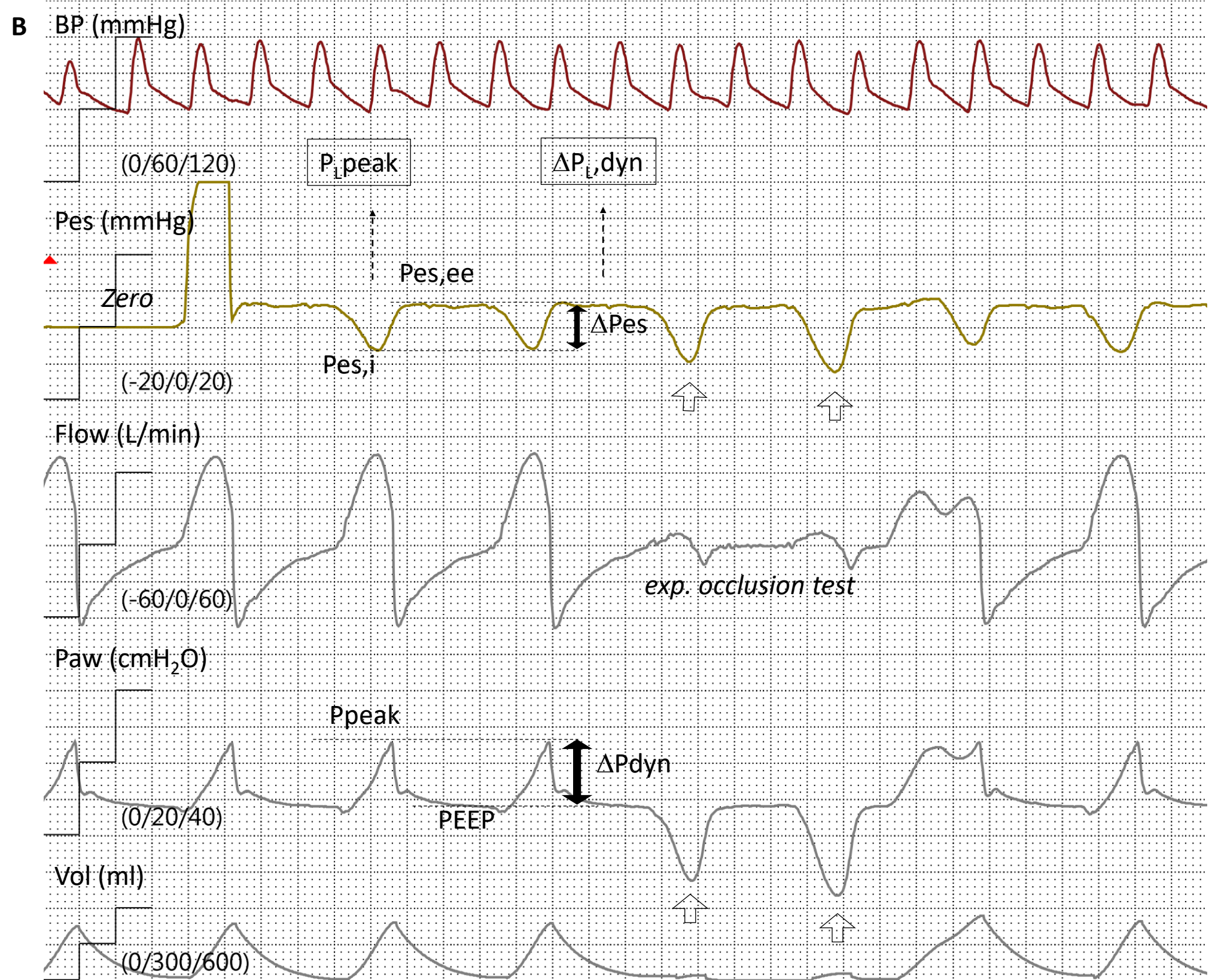

Supplement: Supplementary file 3 — Additional file 3: Figure S3. Air-filled esophageal catheter-guided ventilation in controlled and assisted modes. A. In passive condition, illustrative waveforms of blood pressure (BP), esophageal pressure (Pes, in mmHg), air flow, airway pressure (Paw, in cmH2O) and volume (Vol). After zeroing and subsequent end-inspiratory and end-expiratory occlusions, three sternal compressions (white arrows) induce equivalent increases in esophageal and airway pressures. B. Same waveforms in active condition. In spontaneous breathing, dynamic end-expiratory occlusion test induces two equivalent esophageal and airway depressions (white arrows). ΔP driving pressure, ΔPdyn dynamic driving pressure, ΔPes esophageal pressure swing, ΔPL driving transpulmonary pressure, ΔPLdyn dynamic transpulmonary pressure swing, ECG electrocardiogram, PEEP positive end-expiratory pressure, PEEPtot total PEEP, Pes,ee end-expiratory Pes, Pes,i inspiratory Pes, Pes,plat plateau Pes, PLee end-expiratory transpulmonary pressure, PLei,ER elastance-derived end-inspiratory transpulmonary pressure, PLpeak peak transpulmonary pressure, Ppeak peak airway pressure, Pplat plateau airway pressure. [file 40635_2021_411_MOESM3_ESM.pdf]

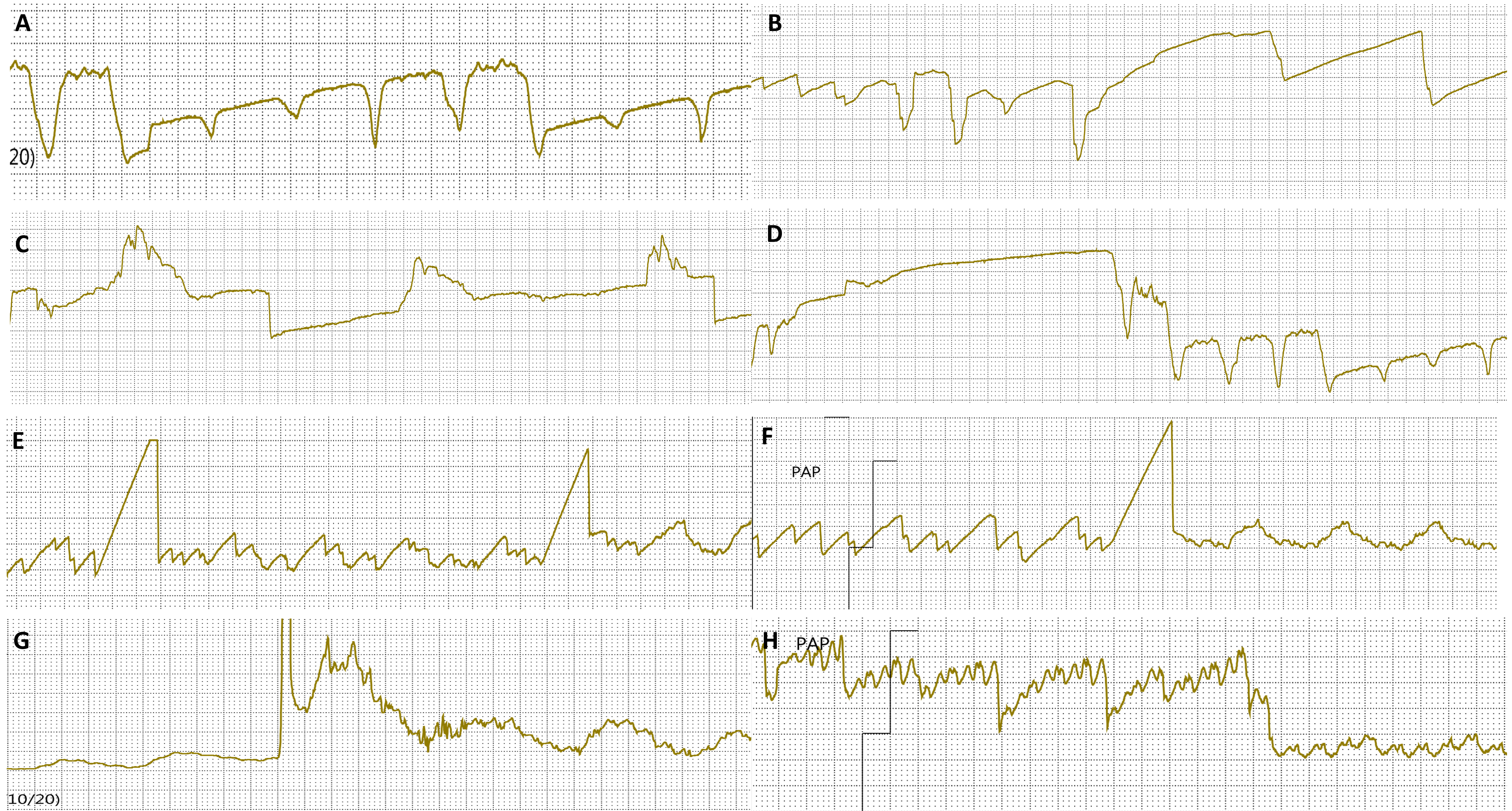

Supplement: Supplementary file 4 — Additional file 4: Figure S4. Subocclusion of the air-filled esophageal catheter and flushing procedure. Abrupt vertical falls, staircase steps or increasing slopes in the esophageal pressure wave (A to D) indicate subocclusion by secretions. Flushing 3 ml of air (E, F) or 10 ml of air (G) or pulling out for 2 cm (H) enables deobstruction in most cases. [file 40635_2021_411_MOESM4_ESM.pdf]

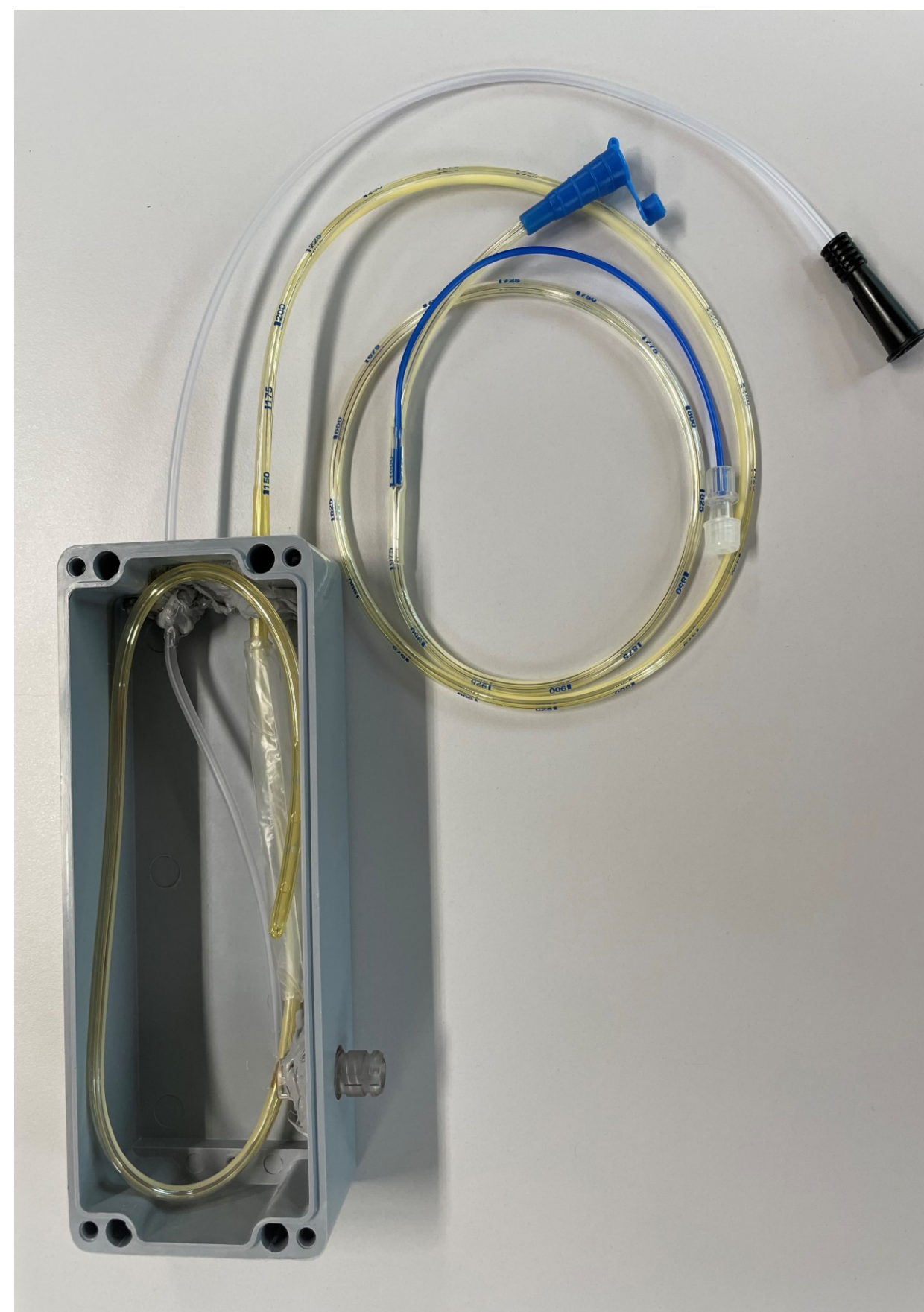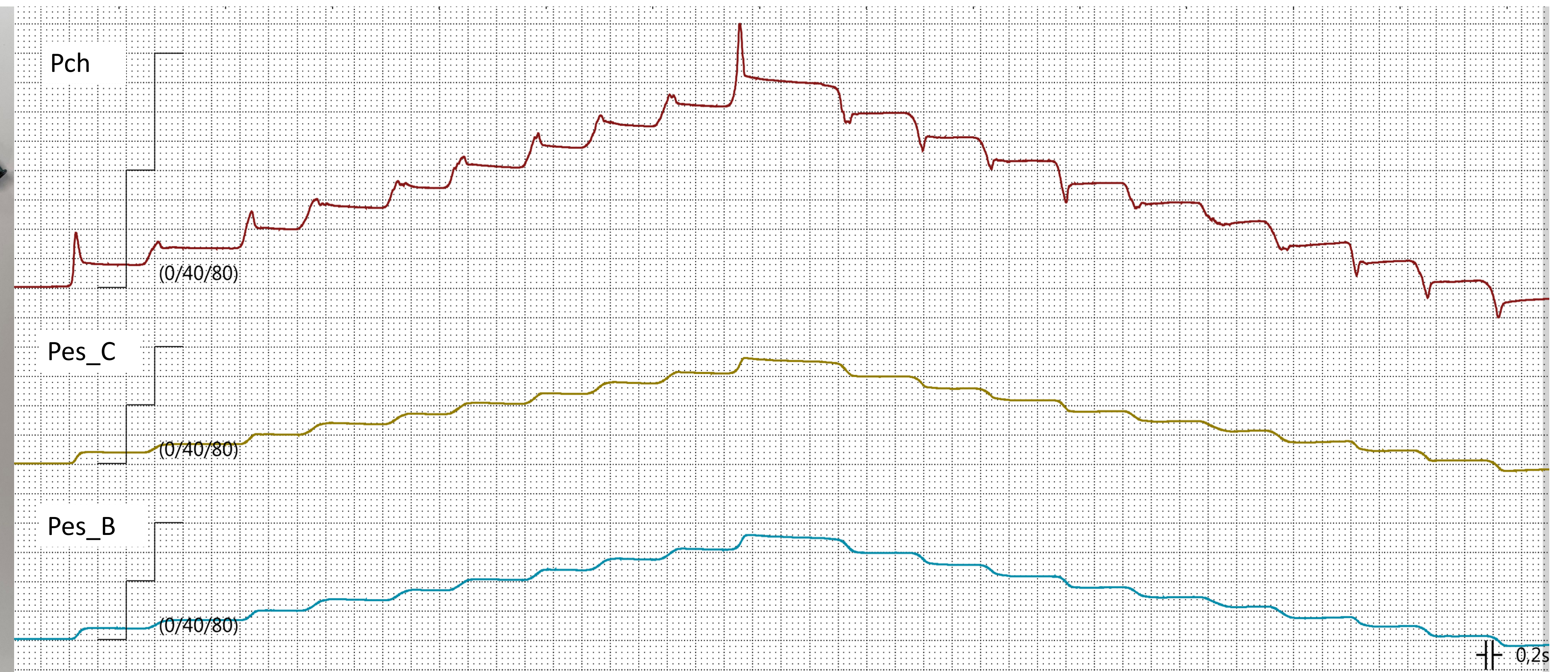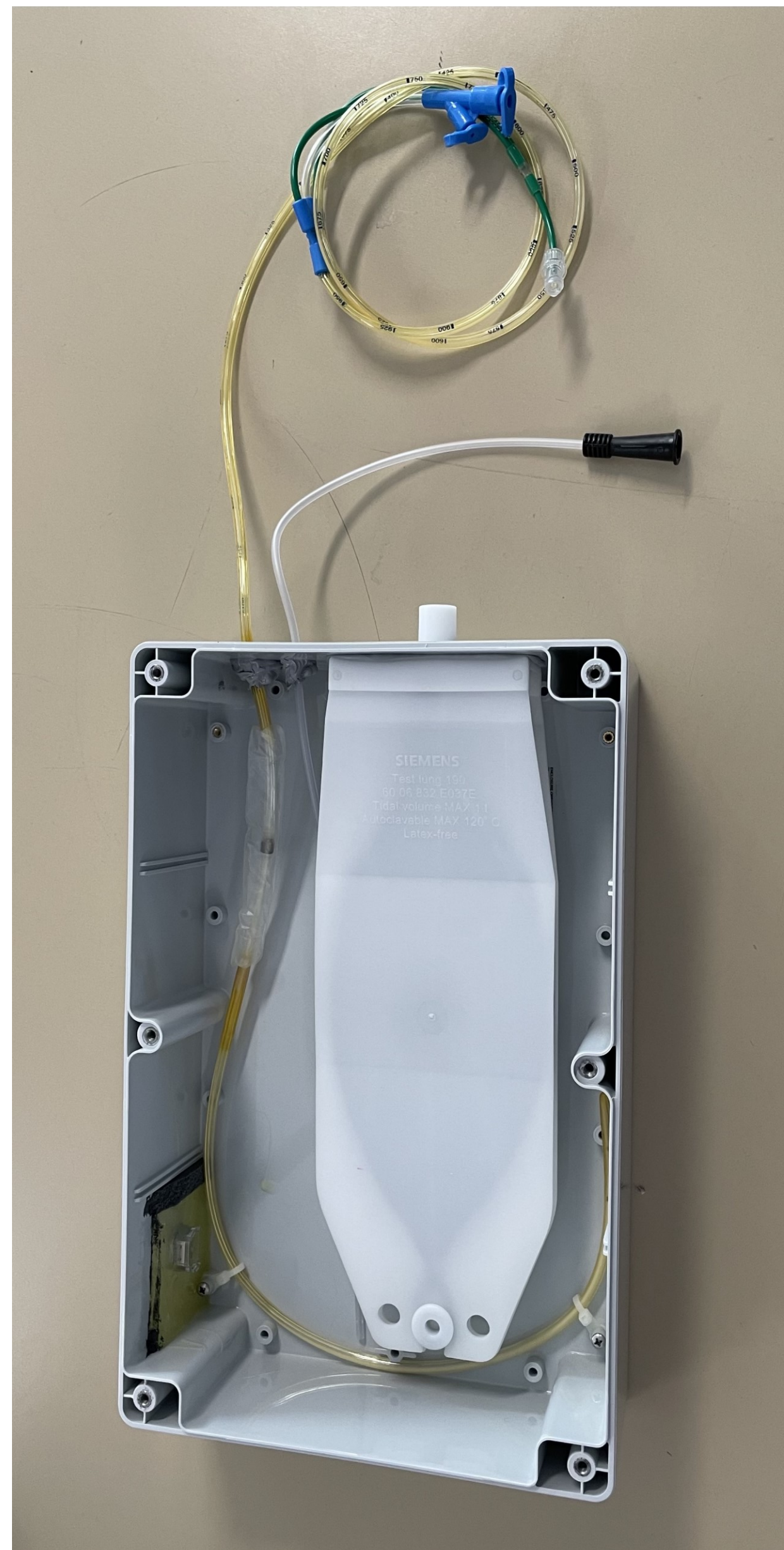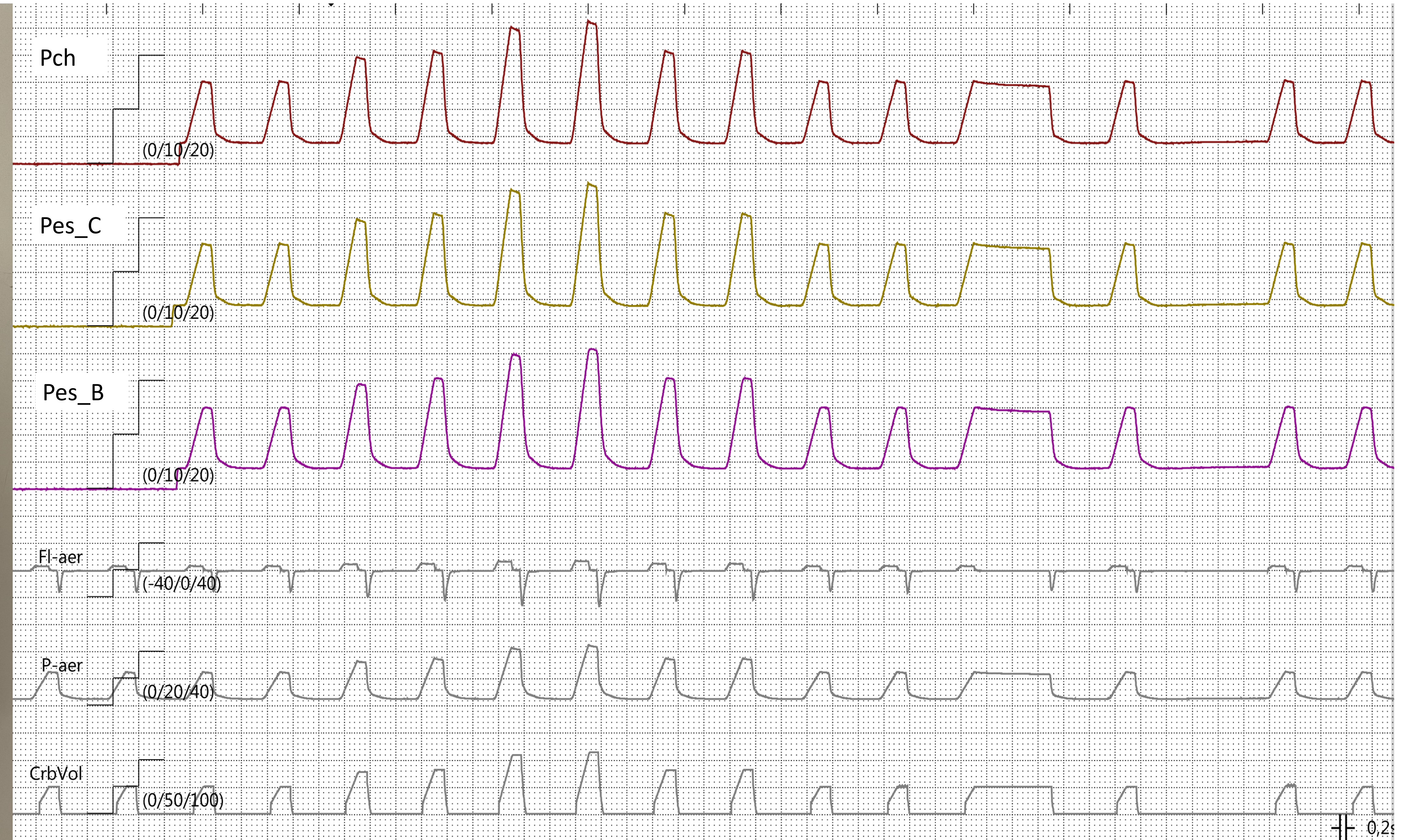

Supplement: Supplementary file 5 — Additional file 5: Figure S5. Ex vivo comparison of air-filled catheter and balloon catheter pressure transductions. A. Ex vivo comparison of air-filled catheter pressure (Pes_C), balloon catheter pressure using a 4 ml balloon volume (Pes_B) and inner chamber pressure (Pch) during 5 ml of air increments from 0 to 50 ml, injected and then removed by the chamber port (right) of the small pressure chamber (left). B. Ex vivo comparison of the same pressures (right), in a larger pressure chamber containing a test lung (left) allowing for 100 ml to 200 ml tidal volume inflations. Two successive (end-inspiratory and end-expiratory) pauses are performed. Note that volume curves underestimate true volume inflation due to tubing distension. [file 40635_2021_411_MOESM5_ESM.pdf]

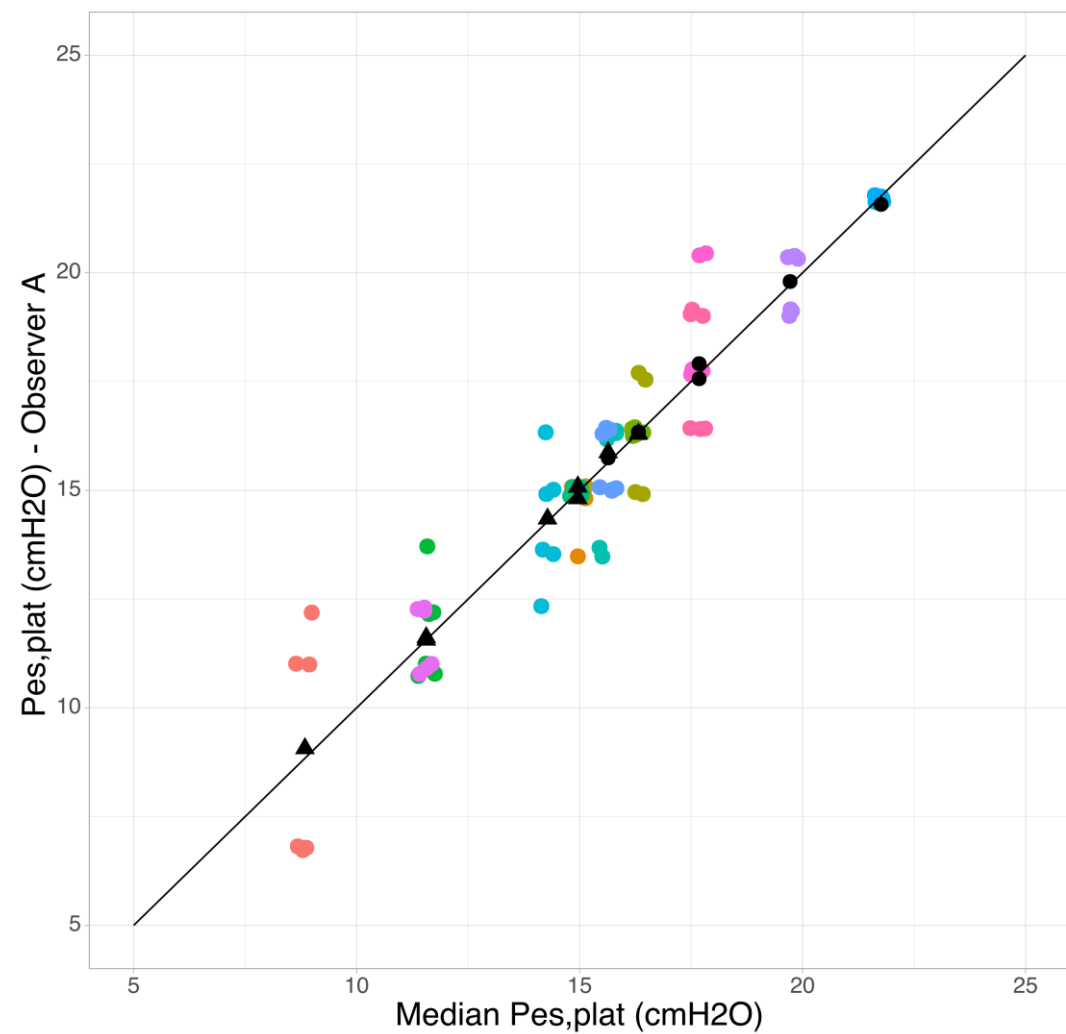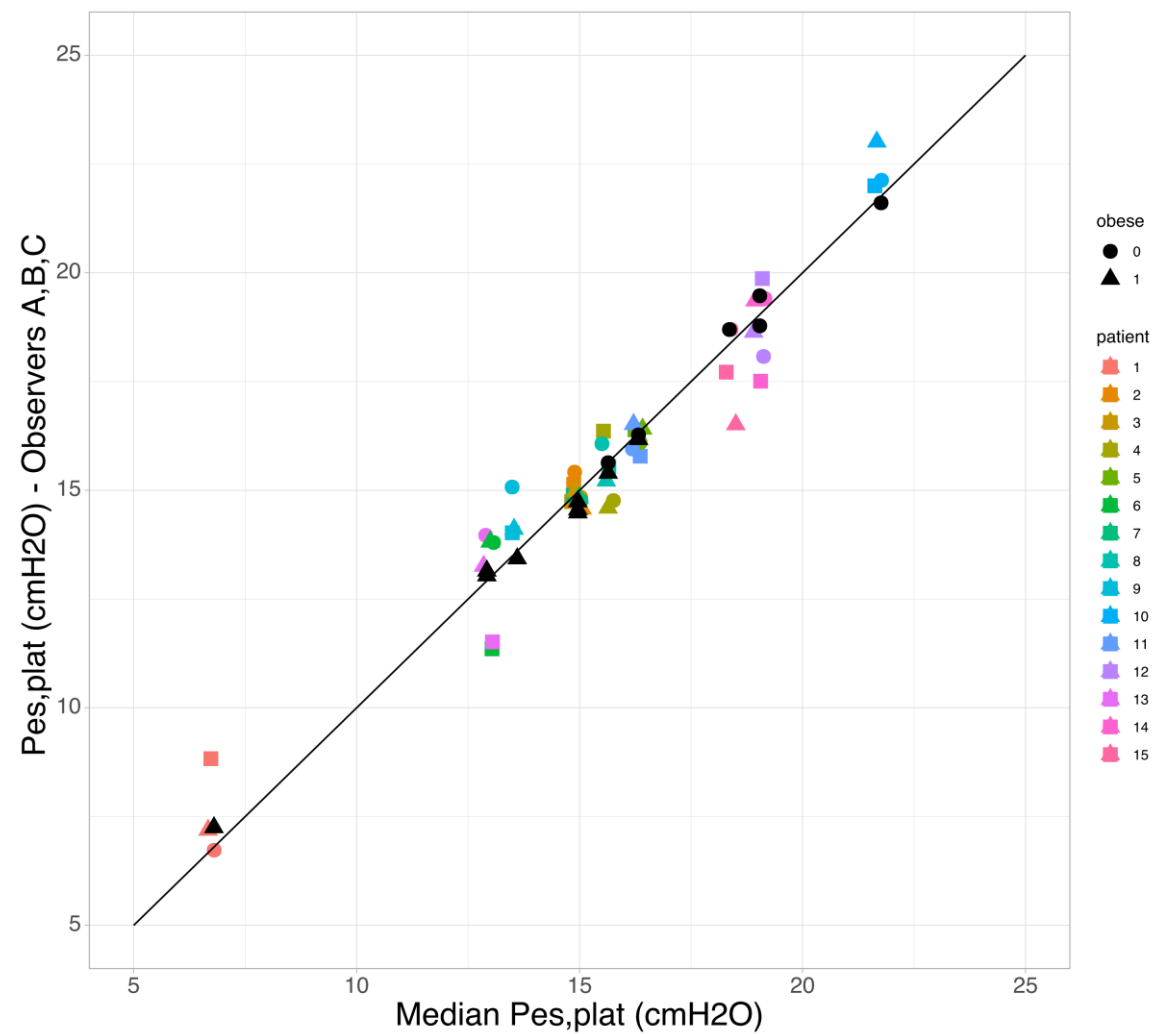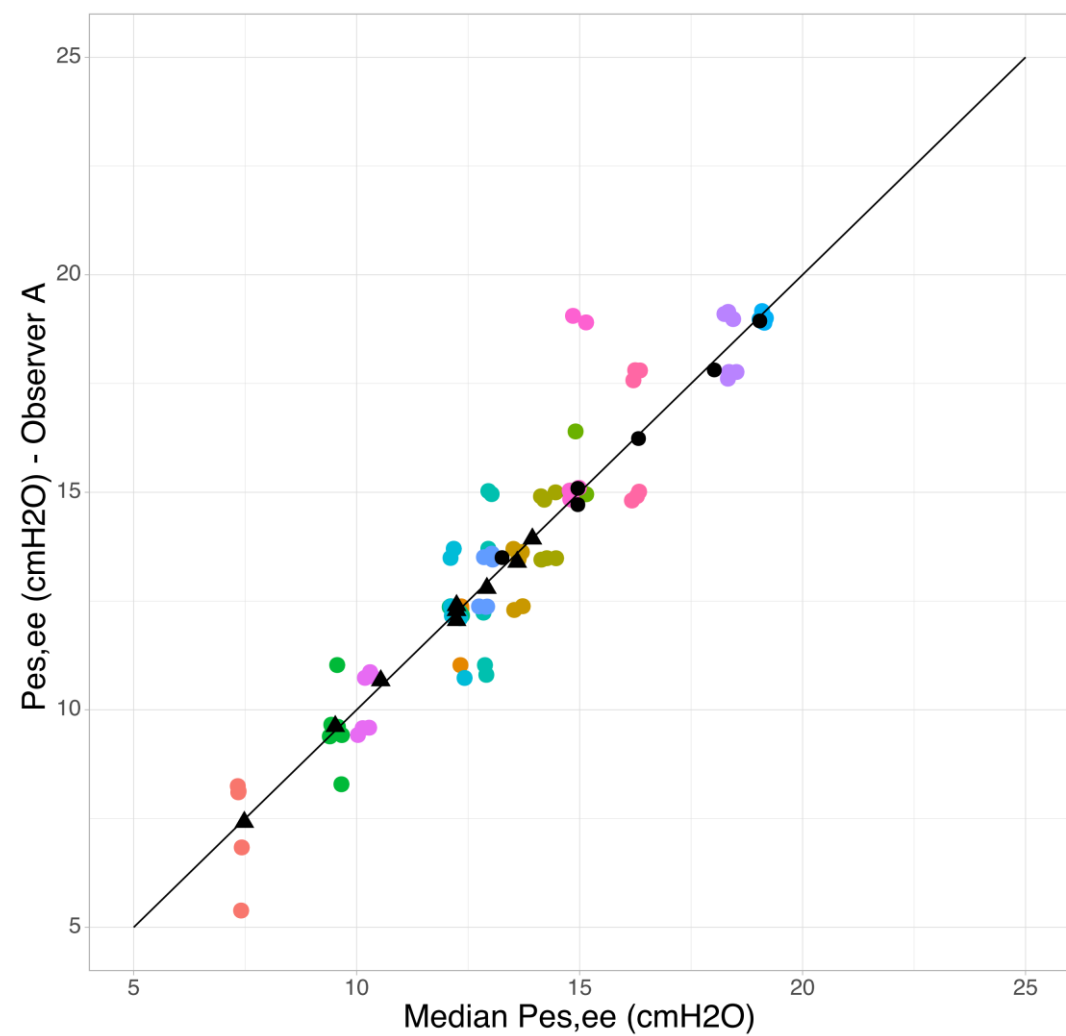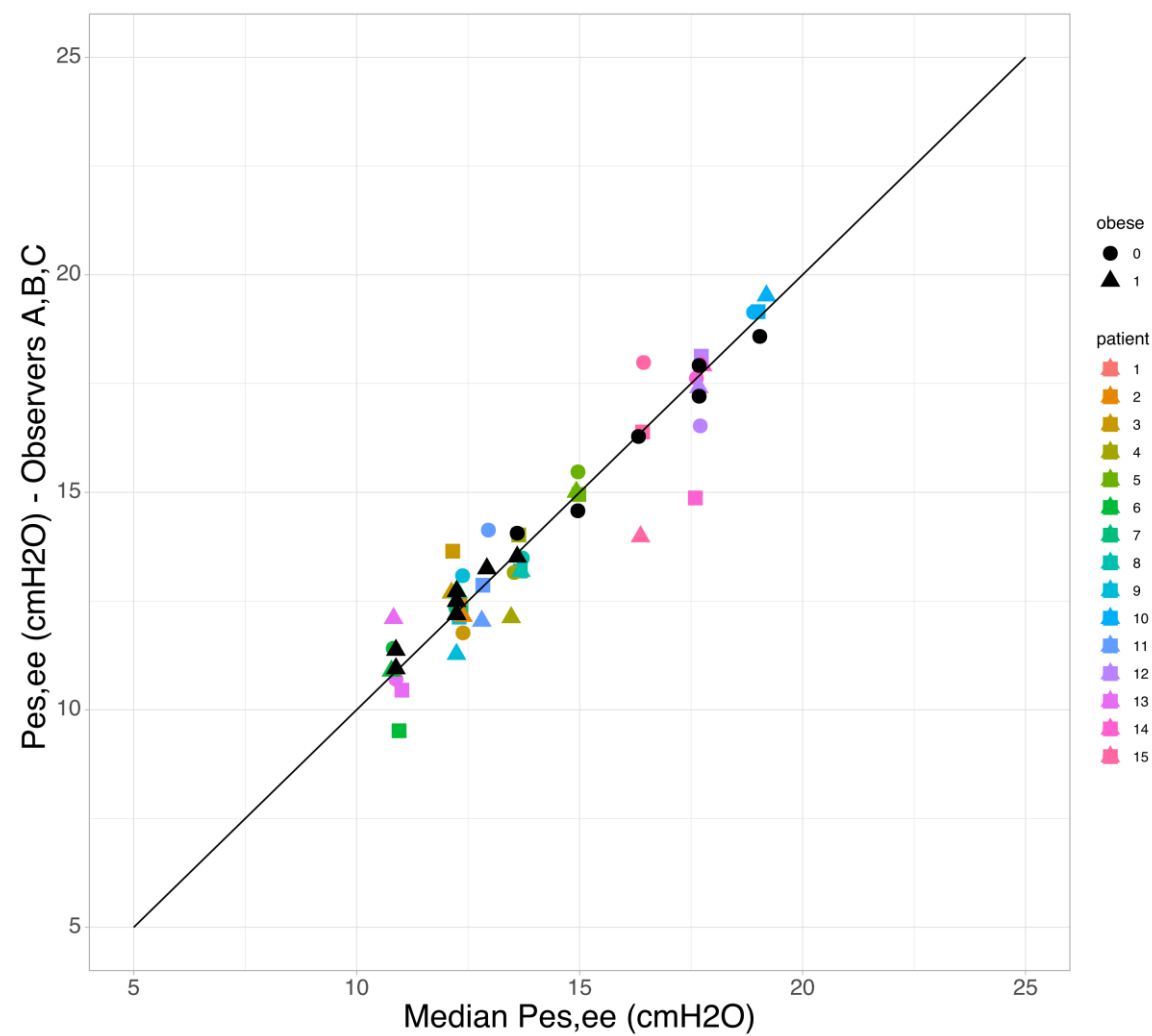

Supplement: Supplementary file 6 — Additional file 6: Figure S6. Repeatability and reproducibility of air-filled esophageal catheter measurements. Variability of plateau (upper panel) and end-expiratory (lower panel) esophageal pressures measured using the air-filled catheter in 15 patients under volume-controlled ventilation. Left, intra-observer variability (repeatability) showing six repeated measurements (colored) by the same observer plotted against their medians (black). Right, inter-observer variability (reproducibility) showing medians from three different observers A, B and C (colored) plotted against the medians from all observers (in black: triangles for non-obese and circles for obese). Lines of equality are shown. [file 40635_2021_411_MOESM6_ESM.pdf]

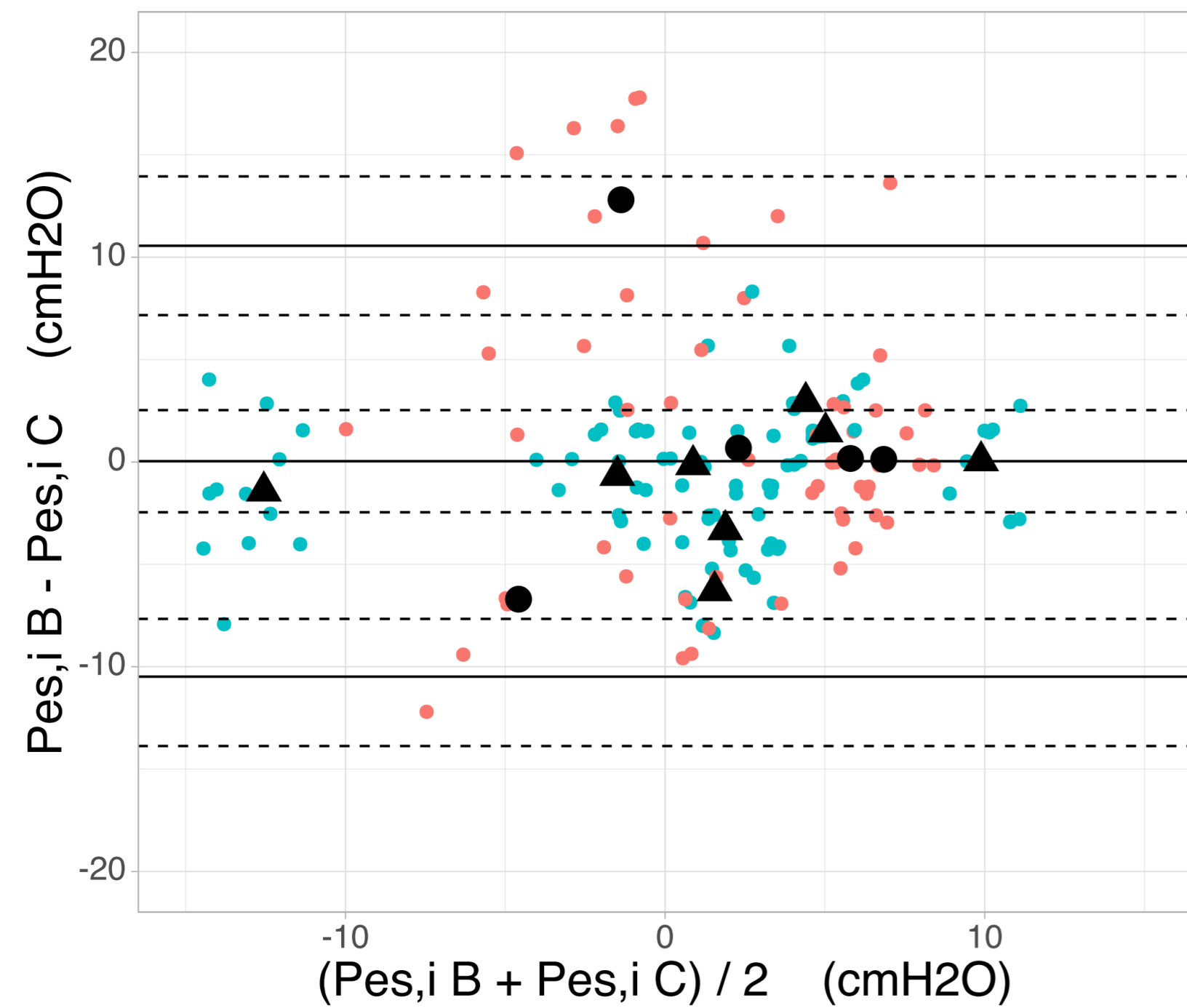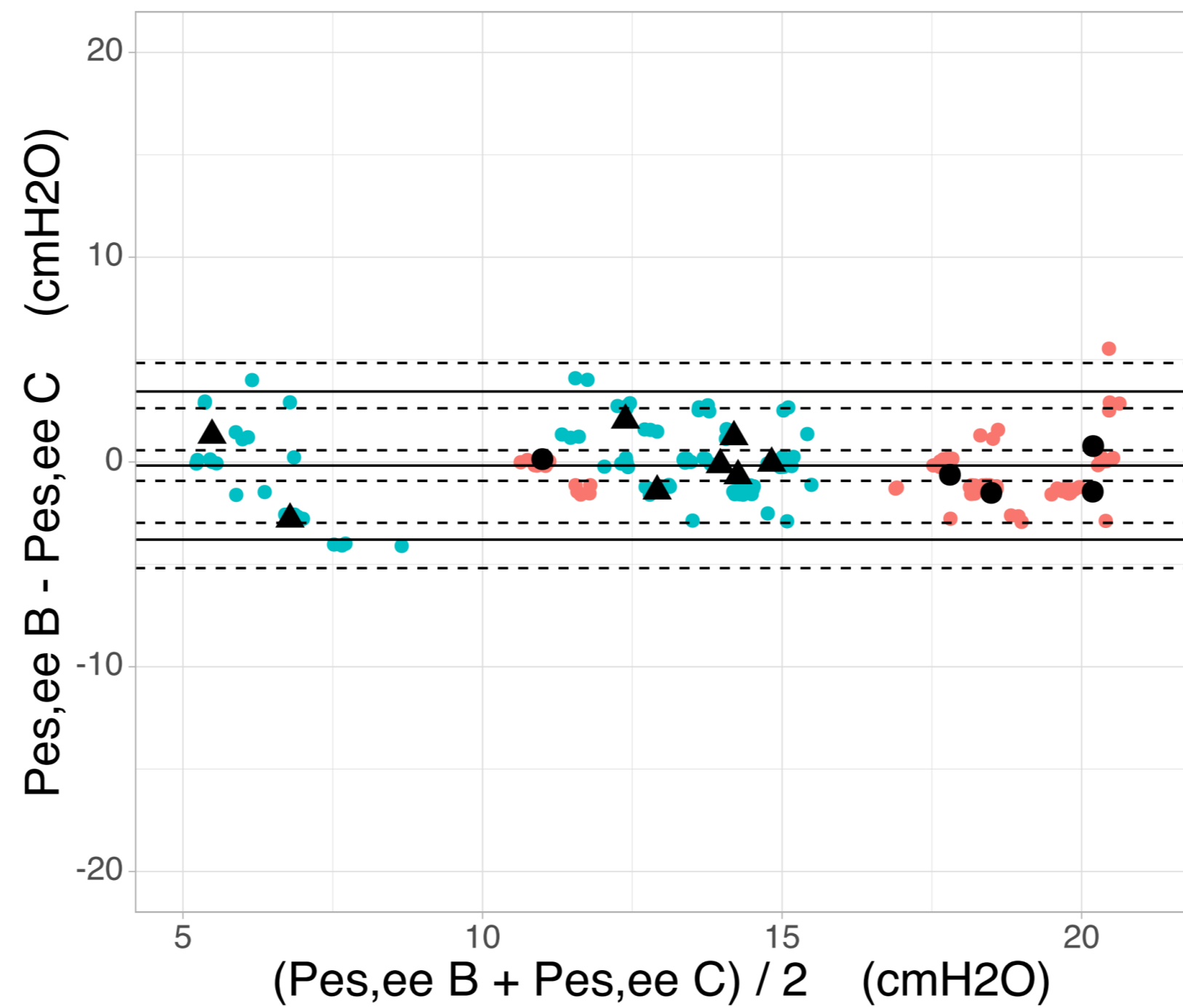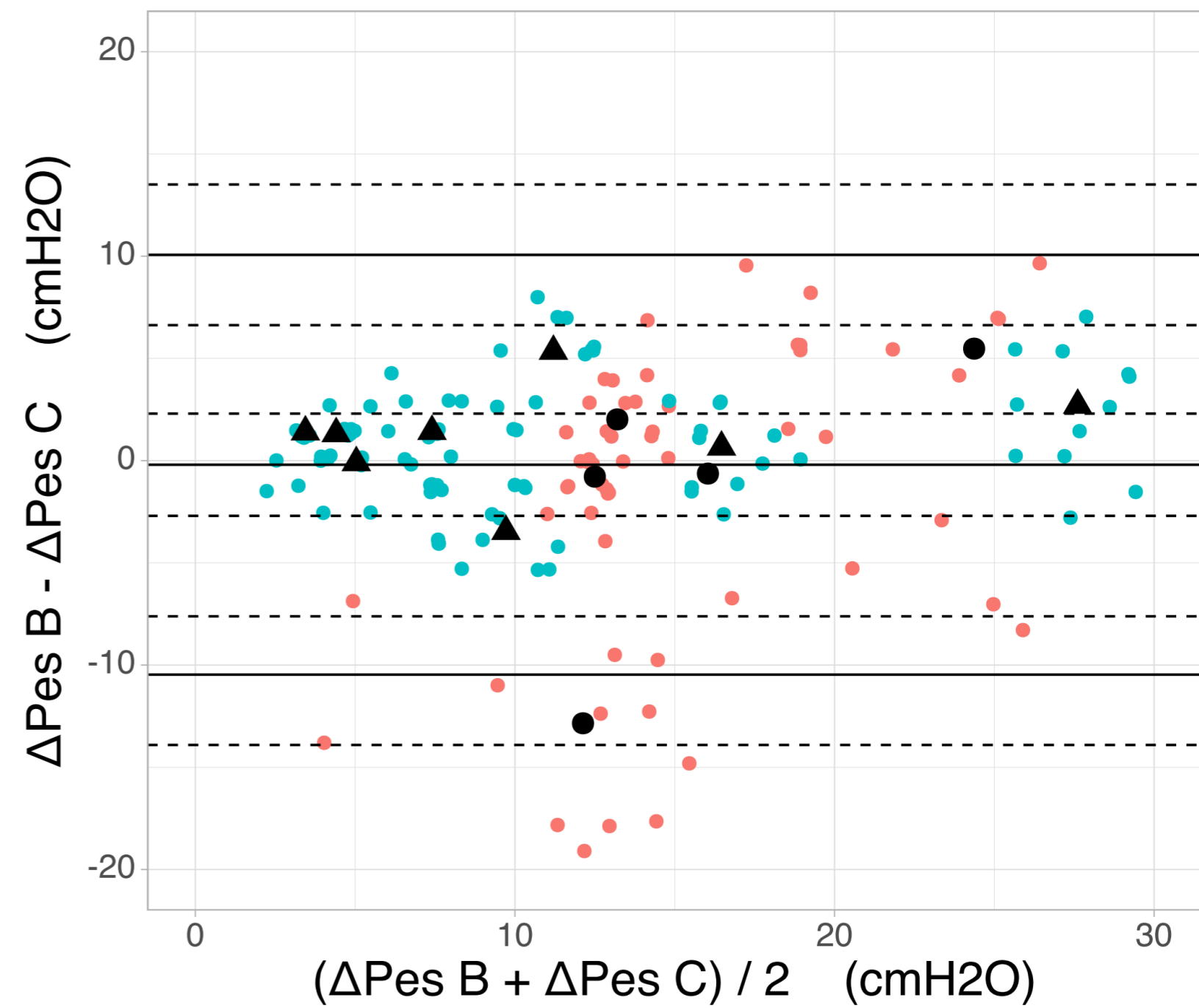

Supplement: Supplementary file 7 — Additional file 7: Figure S7. Bland–Altman analysis in patients under assisted mode. The difference of the peak (left), end-expiratory (middle) and delta (right) esophageal pressure measurements by the air-filled catheter and the balloon methods in assisted mode are plotted against the mean of the measurements. Solid lines represent the mean differences and the limits of agreement. Dashed lines represent their respective 95% confidence interval. The colored circles represent single measurements (n = 12 for each patient), with non-obese patients (n = 8) in green and obese ones (n = 5) in red. Black triangles and circles represent the medians of all measurements in non-obese and obese patients, respectively. [file 40635_2021_411_MOESM7_ESM.pdf]

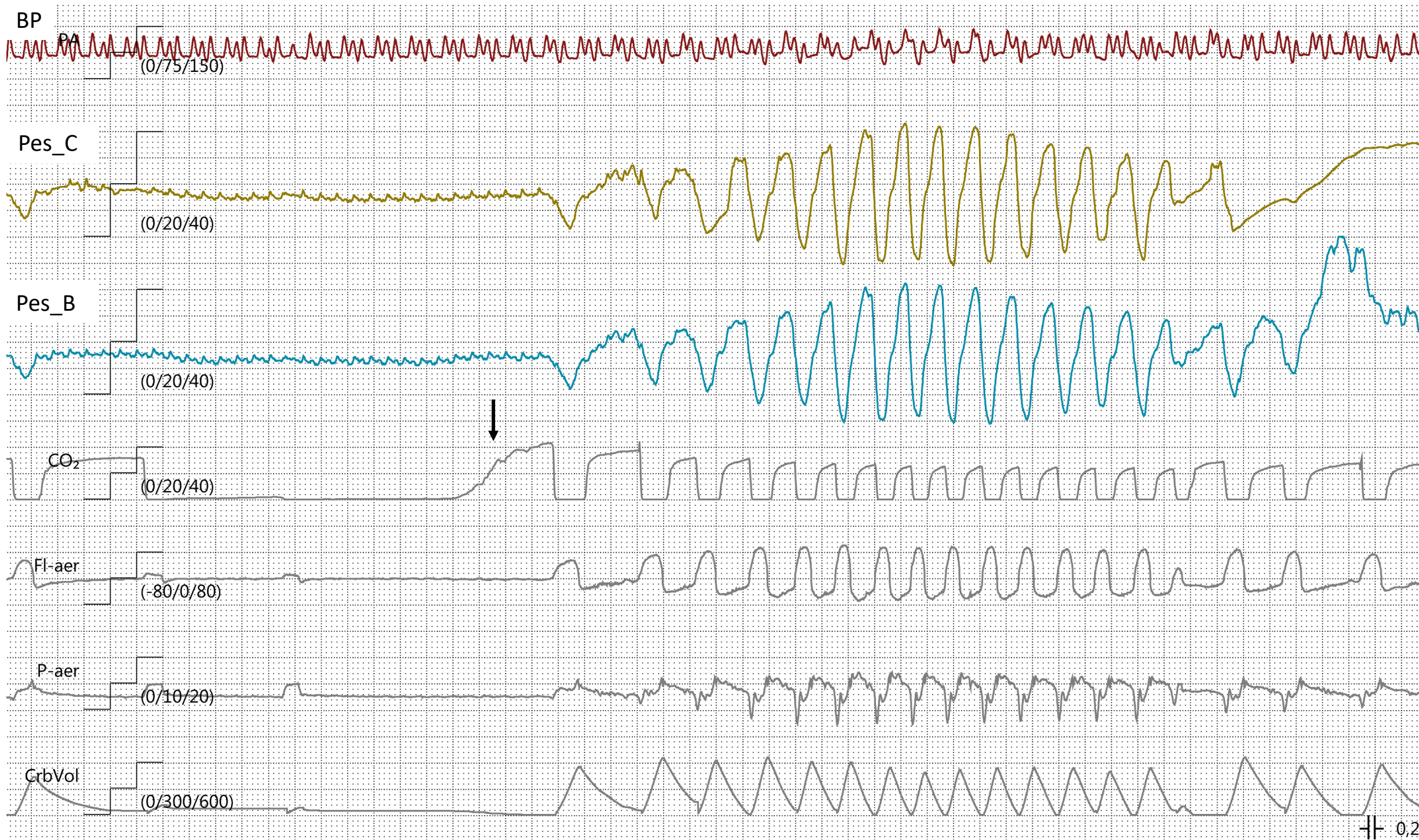

0.2

Supplement: Supplementary file 8 — Additional file 8: Figure S8. Esophageal pressures curves during Cheyne–Stokes respiration. Simultaneous esophageal pressures are recorded with the air-filled esophageal catheter and the balloon catheter in one patient with spontaneous Cheyne–Stokes respiration. Note the increase in expired CO2 before starting polypnea in the lower panel due to active expiration at the end of the respiratory pause (arrow). [file 40635_2021_411_MOESM8_ESM.pdf]
