# Supplementary material for: Novel method of transpulmonary pressure measurement with an air-filled esophageal catheter
Source: Intensive Care Med Exp. 2021 Sep 17;9:47. doi: 10.1186/s40635-021-00411-w (PMC8445653; doi:10.1186/s40635-021-00411-w)
Supplement: Supplementary file 9 — Additional file 9. The complete Standard Operating Procedure of the air-filled esophageal catheter method and the ex vivo comparison of both esophageal pressure methods are presented. [file 40635_2021_411_MOESM9_ESM.docx]

Additional file 9 ICMX-D-21-00008R1

1. **Standard Operating Procedure of the air-filled esophageal catheter method**

- Materials of the air-filled esophageal catheter method:

Here we use a disposable low compliance polyvinyl esophageal suction catheter Mülly with Funnel, originally intended for oral, nasopharyngeal or tracheobronchial suctioning, 49 cm long, 10 Fr and 3.3-2.0 mm outer-inner diameters. As shown in Figure 1, the catheter is connected via a Luer-lock transfer connector to an air-filled disposable blood pressure transducer bound to the monitor. The transducer is identical to the one intended for blood pressure monitoring and contains an integral flush device delivering a continuous flow to maintain catheter patency. A one-liter saline infusion bag is carefully emptied, free of any residual droplets, backfilled with air using a 50ml syringe (Figure S1A) and pressurized at 100 mmHg by a 1000ml reusable pressure infusion bag with manometer (Figure S1B, S1C). The bag is then connected to the air-filled intravenous set with drip chamber and roller clamp, so that the transducer delivers a resulting continuous air flow rate of ~2.5 ml/min for the 10fr catheter. The pressurized system guarantees open-ended catheter patency, undamped values and signal stability. Air-labeled flags are disposed along the air-filled pressure line for safety.

- Placement of the esophageal catheter:

To facilitate the nasal or oral placement of the esophageal catheter and its visualization on chest X-rays, a siliconized guide wire of nasogastric enteral feeding tube is temporarily inserted in the catheter and bended to match the desired length. The catheter follows the naso- or orogastric feeding tube, if present, until its extremity is positioned first in the stomach with its funnel part close to the nostrils. Proper gastric position is assessed by auscultation of a 10 mL air flush and, after connection to the transducer and opening of the roller clamp on the infusion set, by observation of positive deflections on waveform during inspiration or when gentle stomach compressions are imposed. The catheter is withdrawn to the lower third of the esophagus until the length of its inner part at the nostril equals the nasal-tragus-xyphoid distance (~ 50 cm) minus 10 cm (thus ~ 40cm). The resulting length of the outer part of the catheter from nostril to funnel equals the catheter length (49cm) minus its inner part (thus ~ 9cm). The catheter is withdrawn until esophageal waveform is confirmed by small cardiac artifacts and spontaneous inspiratory negative deflections. Appropriate position of the catheter is confirmed in three ways, i) by chest X-rays with the guide wire (Figure S1), ii) by visualization of cardiac artefacts on the esophageal waveform, and iii) by equivalent changes in esophageal and airway pressures during the dynamic end-expiratory occlusion test maneuver. In passive breathing condition, gentle external chest compressions are performed during expiratory occlusion. In active breathing condition, spontaneous efforts occur against occlusion (Baydur’s maneuver). Esophageal to airway pressure change ratio (ΔPes/ΔPaw) should be close to one (± 10-20%).

- Esophageal pressure measurements:

Before any esophageal measurements, the open-ended catheter is flushed with 3ml of air using a syringe in order to remove any distal secretion. Residual esophageal air is then aspirated until there is a backward movement of the syringe plunger, to prevent potential artifact leading to an overestimation of the measurement, in a very gentle manner to avoid re-aspiration of secretions. The line is left open to air to equilibrate for a few seconds and then is turned for reading. If the esophageal pressure trace is suboptimal, one more ml of air is injected to allow optimal pressure transduction. We record the zero with the transducer open to atmospheric pressure. Values are recorded in mmHg and converted in cmH_2_O.

Esophageal catheter subocclusion by secretions is suspected when abrupt, vertical falls, staircase steps or increasing slopes disrupt the esophageal pressure wave. Deobstruction of the catheter requires the following flushing procedure to restore a proper signal: flushing 3ml of air, twice if needed; if unsuccessful, pushing the catheter with its benched guidewire lower to the stomach for a new flush of 10 ml of air before repositioning it in the esophagus; if failure, replacement of the catheter. Placement of the new esophageal catheter follows the following simplified procedure: with the benched guidewire, the catheter is lowered along the feeding tube to the stomach with the funnel at the nostrils, flushed with air to confirm stomach position, then pulled up into the esophagus at the already-known adequate distance from the nostril and connected to the circuit to see cardiac artefacts.

- Transpulmonary pressure-guided lung-protective ventilation:

For clinical purpose, we measure end-inspiratory and end-expiratory pressures in both airway and esophagus, during controlled and assisted modes. This allows computation of transpulmonary pressures in order to apply an optimal transpulmonary-guided lung-protective ventilation.

Theoretically, esophageal pressure measurements may help clinicians to individualize lung-protective ventilation. In passive breathing condition (Figure S2A), end-inspiratory and end-expiratory occlusions indicate plateau airway pressure (Pplat), plateau esophageal pressure (Pes,plat), total positive end-expiratory pressure (PEEPtot), end-expiratory esophageal pressure (Pes,ee), driving pressure ΔP (ΔP = (Pplat – PEEPtot)) and delta esophageal pressure ΔPes (ΔPes = (Pes,plat – Pes,ee)). Ecw (Ecw = ΔPes/Vt) is calculated as the ratio of ΔPes to Vt, El (El = Ers – Ecw) is obtained by subtracting Ecw from respiratory system elastance (Ers; Ers = ΔP/Vt). Elastance ratio (ER; ER = El/Ers) is the lung to respiratory system elastance ratio. Three transpulmonary pressures are calculated, with potential therapeutic targets [1, 14]: i) elastance-derived end-inspiratory P_L_ (P_L_ei,_ER_; P_L_ei,_ER_ = Pplat x El/Ers) < 20 cmH_2_O; ii) end-expiratory P_L_ (P_L_ee; P_L_ee = (PEEPtot – Pes,ee)) > 0 cmH_2_O; iii) driving P_L_ (ΔP_L_; ΔP_L_ = (ΔP - ΔPes) = (P_L_plat – P_L_ee)) < 10-12 cmH_2_O.

In active breathing condition (Figure S2B), semi-recumbent patients are ventilated in pressure support mode with spontaneous inspiratory efforts. Peak airway pressure (Ppeak), inspiratory esophageal pressure (Pes,i), PEEP, end-expiratory esophageal pressure (Pes,ee), ΔPes (ΔPes = (Pes,i – Pes,ee)) and dynamic airway driving pressure (ΔPdyn; ΔPdyn = (Ppeak – PEEP)) values are measured. Three transpulmonary pressures are also calculated, with potential therapeutic targets [1, 14]: peak P_L_ (P_L_peak; P_L_peak = (Ppeak – Pes,i)) < 20 cmH_2_O, ΔPes < -10 to -15 cmH_2_O and dynamic P_L_ swing (ΔP_L_dyn; ΔP_L_dyn = (ΔPdyn – ΔPes)) < 15 cmH_2_O.

To facilitate bedside calculations, we have designed an online transpulmonary pressures calculator accessible at [www.esophageal-pressure-calculator.be](http://www.esophageal-pressure-calculator.be) [17].

1. **Ex vivo comparison of both esophageal pressure methods**:

We performed simultaneous air pressure measurements via the air-filled catheter and the balloon catheter placed in two airtight pressure chambers (Figure S5). A first small 527 ml pressure chamber recorded a 10-step 5 ml increment test from 0 to 50 ml injected in the chamber by a syringe. A second larger 4400 ml pressure chamber including one 1L test lung allowed mechanical ventilation with 100-150-200 ml tidal volume inflations (PEEP 5 cmH_2_O, respiratory rate 25) to better mimic the patient’s condition.
